# Benzothiadiazole vs. *iso*-Benzothiadiazole: Synthesis, Electrochemical and Optical Properties of D–A–D Conjugated Molecules Based on Them

**DOI:** 10.3390/molecules26164931

**Published:** 2021-08-14

**Authors:** Nikita S. Gudim, Ekaterina A. Knyazeva, Ludmila V. Mikhalchenko, Ivan S. Golovanov, Vadim V. Popov, Natalia V. Obruchnikova, Oleg A. Rakitin

**Affiliations:** 1 N. D. Zelinsky Institute of Organic Chemistry, Russian Academy of Sciences, 119991 Moscow, Russia; nikitosgudim@gmail.com (N.S.G.); katerina_knyazev@ioc.ac.ru (E.A.K.); mlv@ioc.ac.ru (L.V.M.); cell-25@yandex.ru (I.S.G.); dlt@ioc.ac.ru (N.V.O.); 2 Nanotechnology Education and Research Center, South Ural State University, 454080 Chelyabinsk, Russia; popov.ioc@gmail.com

**Keywords:** benzo[*d*][1,2,3]thiadiazole, benzo[*c*][1,2,5]thiadiazole, π-spacer–acceptor–π-spacer type structures, cross-coupling reactions, optical properties, electrochemical properties

## Abstract

This paper presents an improved synthesis of 4,7-dibromobenzo[*d*][1,2,3]thiadiazole from commercially available reagents. According to quantum-mechanical calculations, benzo[*d*][1,2,3]thiadiazole (isoBTD) has higher values of E_LUMO_ and energy band gap (E_g_), which indicates high electron conductivity, occurring due to the high stability of the molecule in the excited state. We studied the cross-coupling reactions of this dibromide and found that the highest yields of π-spacer–acceptor–π-spacer type compounds were obtained by means of the Stille reaction. Therefore, 6 new structures of this type have been synthesized. A detailed study of the optical and electrochemical properties of the obtained π-spacer–acceptor–π-spacer type compounds in comparison with isomeric structures based on benzo[*c*][1,2,5]thiadiazole (BTD) showed a red shift of absorption maxima with lower absorptive and luminescent capacity. However, the addition of the 2,2′-bithiophene fragment as a π-spacer resulted in an unexpected increase of the extinction coefficient in the UV/vis spectra along with a blue shift of both absorption maxima for the isoBTD-based compound as compared to the BTD-based compound. Thus, a thorough selection of components in the designing of appropriate compounds with benzo[*d*][1,2,3]thiadiazole as an internal acceptor can lead to promising photovoltaic materials.

## 1. Introduction

Benzo[*c*][1,2,5]thiadiazole (BTD) appears to be one of the most widely used acceptor heterocycles in producing donor-acceptor materials for organic electronics [1,2,3,4]. The symmetric structures, in which the 4 and 7 positions of the benzothiadiazole ring are replaced by identical substituents, are widely used as monomers for the obtaining of donor-acceptor polymers, and for the synthesis of small molecules with various useful photovoltaic properties [5,6,7,8]. The strong electron-acceptor properties of BTD provide materials based on it with the necessary properties (band gap, charge transport properties, intra- and intermolecular interactions, etc.), which can be fine-tuned by combining BTD with various donor or π-conjugated systems. However, over several decades of active study, the vast possibilities of this heterocyclic system have been almost fully exhausted. In this regard, it becomes relevant to study the closest analogue of BTD, benzo[*d*][1,2,3]thiadiazole (isoBTD), its isomer, which differs only by the order of atoms in the five-membered ring. It should be noted that isoBTD is practically unstudied as a building block. Only few reports devoted to its synthesis and some reactions are known. This is even more astonishing since its key derivative for the synthesis of various photovoltaic materials, 4,7-dibromobenzo[*d*][1,2,3]thiadiazole, is easily obtained in two steps using the commercially available 2-aminobenzothiol [9]. Its chemical transformations are limited to the Stille reaction with 2-tributylstannyl thiophene, Stille co-polymerization [9], and nucleophilic substitution with morpholine [10].

**Figure 1:**
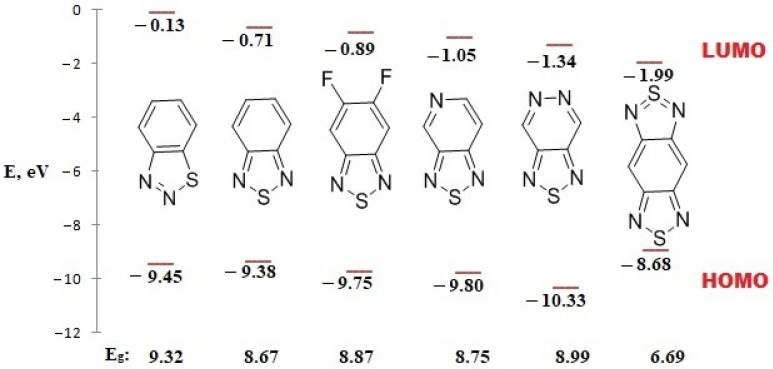
HOMO-LUMO energy levels and energy gap values for electron deficient heterocycles with thiadiazole motifs.

The success of the BTD fragment as a building block for donor-acceptor materials lies in its high electron affinity, which determines the energy of the lowest unoccupied molecular orbital (E_LUMO_). Various methods are known to decrease the LUMO energy of the acceptors by adding electronegative groups, such as fluorine [11,12]; by using conjugated electron-deficient heterocycles [13,14]; and by replacing carbon atoms in aromatic systems with more electronegative atoms, such as nitrogen [15,16]. Figure 1 shows that application of the above described approaches leads to a reduction of E_LUMO_ from −0.71 eV for BTD to −0.89 eV for 5,6-difluorobenzo[*c*][1,2,5]thiadiazole and −1.99 eV for benzo[1,2-*c*:4,5-c′]bis[1,2,5]thiadiazole. However, as we showed earlier by the example of condensation of 1,2,5-thiadiazole with more electron acceptor cycles than benzene (pyridine [15,17] and pyridazine [16,18]), a significant decrease of E_LUMO_ (to −1.05 eV and −1.34 eV, respectively) is not always the key to success in producing highly efficient materials. According to the calculated data, the results of which are shown in Figure 1, isoBTD has a higher LUMO energy than its well-studied isomer, but enough to provide electron affinity.

Another important characteristic that determines the efficiency of photoelectronic materials is the energy band gap (E_g_), the value of which determines the stability of the molecule in the excited state. The E_g_ value for this proposed new internal acceptor is the highest among those heterocycles which electron-acceptor properties have been increased by one of the methods described above. This indicates high electron conductivity, which results from the high stability of the molecule in the excited state. Such a difference in the energy profile of the two isomers allows us to hope that compounds based on the poorly studied isoBTD will exhibit properties close to BTD, on the one hand, and compare favorably with it, on the other.

4,7-Diaryl(hetaryl)-disubstituted benzo[*c*][1,2,5]thiadiazoles of the π–A–π type are among the most studied derivatives of this heterocycle since they are precursors of various types of photovoltaic materials, including DSSCs, OLEDs and OFETs, both of polymeric and oligomeric structure [2,3,4,5,7]. The most common ways to create a C-C bond between acceptor and donor or π-excess fragments are cross-coupling reactions or the direct C-H arylation between the acceptor dibromo derivatives and the corresponding π-spacer derivatives. Herein, we describe the modified synthesis of 4,7-dibromobenzo[*d*][1,2,3]thiadiazole and their cross-coupling reactions with π-spacers for the preparation of π-spacer–acceptor–π-spacer conjugated molecules which are of interest as photovoltaic materials and study of their electrochemical and optical properties in comparison with corresponding of π–A–π type molecules based on benzo[*c*][1,2,5]thiadiazole.

## 2. Results and Discussion

### 2.1. Synthesis of 4,7-Dibromobenzo[d][1,2,3]thiadiazole

**Scheme 1:**
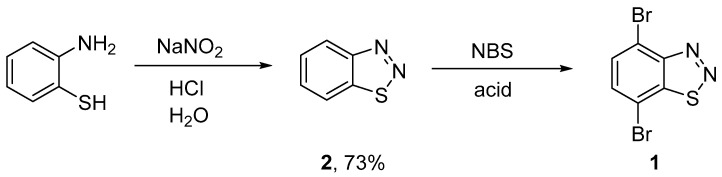
Synthesis of 4,7-dibromobenzo[*d*][1,2,3]thiadiazole **1**.

Despite the fact that 4,7-dibromobenzo[*c*][1,2,5]thiadiazole has been extensively studied for the past 20 years [1,2,3,4,5,6,7,8], its closest isomer, 4,7-dibromobenzo[*d*][1,2,3]thiadiazole **1**, was first synthesized relatively recently in 2016 [9]. The obtaining of dibromide **1**, as previously described [9], involves the bromination reaction of benzo[*d*][1,2,3]thiadiazole **2**, which was obtained using the commercially available 2-aminobenzenethiol with a nitrosation reaction followed by intramolecular cyclization (Scheme 1) [19].

Despite the resemblance of the chemical structures of BTD and isoBTD, for the addition of two bromine atoms to isoBTD the authors [9] used a technique different from the procedure for obtaining 4,7-dibromobenzo[*c*][1,2,5]thiadiazole [20]: instead of bromination with a threefold excess of bromine in hydrobromic acid the authors used bromination of N-bromosuccinimide (NBS) in concentrated sulfuric acid, and the reaction was carried out at a long holding time of 16 h at 50 °C and a low concentration of the initial non-brominated substrate, which led to the need for large amounts of concentrated H_2_SO_4_, which must then be utilized when scaling this reaction. We have set out to find more efficient conditions, for which the bromination reaction of isoBTD NBS in various acids was studied in detail (Table 1).

Replication of the reference conditions made it possible to obtain the target product **1** in a yield comparable to that described earlier [9] (Entry 1, Table 1). A more profound study of the reaction showed that increasing the concentration of the initial compound and decreasing the reaction time while maintaining a high yield is possible by increasing the excess of the brominating agent to twice as much (Entry 3, Table 1). Using an even more concentrated solution (Entry 4, Table 1) and further increasing the excess of NBS (Entry 5, Table 1) did not improve the results. With two-fold excess of the brominating agent and the use of an original dilution of 0.07 M, the reaction is expected to be slower than in the more concentrated solution (compare the yields of **1** in Entries 2 and 3, Table 1).

We have shown that the reaction does not proceed in the absence of acid, or when an acid solution in an organic solvent is used (Entries 6 and 7, Table 1). Replacing sulfuric acid with trifluoroacetic acid also does not lead to the reaction product (Entry 8, Table 1). Still, the use of the stronger trifluoromethanesulfonic acid in undiluted form made it possible to obtain the target dibromide **1** in the highest yield (Entry 9, Table 1). Moreover, this result was achieved using a more concentrated solution of non-brominated isoBTD **2** in acid (0.28 M), which resulted in a 4-fold reduction in acid use.

The mechanism of the bromination of the NBS aromatic ring under acidic conditions is well studied and involves the limiting stage of arene protonation followed by the rapid interaction of the resulting protonated species with NBS with the formation of succinimide [21]. Trifluoroacetic acid, as the weakest of the acids considered, is probably incapable of protonating the initial substrate, while triflic acid, the strongest acid in this range, protonates substrate **2** faster than concentrated sulfuric acid. Increasing the excess of NBS also predictably leads to an increased yield of dibromide **2** in a shorter reaction time.

The optimal reaction conditions found in sulfuric acid were applied to obtain 4,7-dibromobenzo[*d*][1,2,3]thiadiazole on a big scale almost without reducing the yield of the target product (Entry 10, Table 1). The spectral data (^1^H, ^13^C NMR, and HRMS spectra) of **1** are similar to those given in the literature [9]. It is worth specially noting that under conditions of bromination benzo[*c*][1,2,5]thiadiazole (Br_2_ in HBr) its isomer benzo[*d*][1,2,3]thiadiazole **2** turned out to be inert.

### 2.2. Cross-Coupling Reactions of 4,7-Dibromobenzo[d][1,2,3]thiadiazole

The closest analogue of this derivative, 4,7-dibromo[*c*][1,2,5]benzothiadiazole, reacted readily with thiopheneboronic acid, its pinacolate ester, tributylstannyl or even with thiophene in C-H-direct arylation conditions to give the corresponding bis-4,7-thienylderivative in high yields [22].

**Scheme 2:**
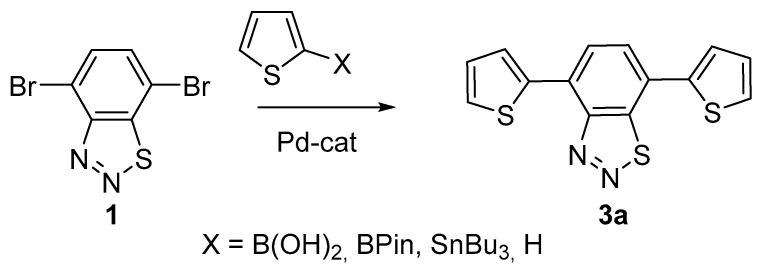
Synthesis of bis-4,7-thienylderivative **3a**.

The behavior of 4,7-dibromobenzo[*d*][1,2,3]thiadiazole **1** under various cross-coupling conditions with thiophene derivatives was studied (Scheme 2). For all reactions, more than double excess of thiophene derivatives was used in order to obtain the bis(thienyl) derivative **3a**. The results of this study are summarized in Table 2.

A study of the Suzuki reaction of 4,7-dibromobenzo[*d*][1,2,3]thiadiazole **1** with thienylboronic acid and with its pinacolate ester showed that the yields of the bis(thienyl) derivative **3a** generally ranged around 50% when using a mixture of solvents that are capable of solubilizing organic substrates (THF, toluene, benzene, or dioxane) and inorganic salts (water, ethanol) (Table 2, Entries 1,4,5,6). When using either one type of solvent (water or toluene), the yield of the resulting compound **3a** decreased sharply to 5–9% (Table 2, Entries 2 and 3). The yields for the pinacolate ester were slightly lower than for the corresponding thiophenboronic acid (compare Entries 1 and 6 in Table 2).

A study of the Stille reaction with 2-tributylstannylthiophene showed that the result of the reaction does not depend much on the applied conditions; the yields in all cases were average (Table 2, Entries 8–13). The most stable results were obtained with the use of PdCl_2_(PPh_3_)_2_ in toluene (Table 2, Entry 8). Noteworthy, the yield we obtained of compound **3a** fully coincides with the literature yield of this compound [9].

Rather unexpected results were obtained when we investigated the direct C-H arylation reaction of 4,7-dibromobenzo[*d*][1,2,3]thiadiazole with thiophene. In the literature, it has been described, that high yields of bis(thienyl) derivatives from 40% to 72% were achieved in this reaction for the BTD analogue and thiophene [23,24]. However, the application of both the conditions described in the reference materials and the recently described conditions with TBAB as quaternary ammonium salt [25] did not lead to success. In all cases 4,7-di(thiophen-2-yl)benzo[*d*][1,2,3]thiadiazole **3a** was formed in low yields.

Thus, the best yield of 4,7-di(thiophen-2-yl)benzo[*d*][1,2,3]thiadiazole **3a** was achieved using the Stille reaction with PdCl_2_(PPh_3_)_2_ as a catalyst in toluene (Table 2, Entry 8).

**Scheme 3:**
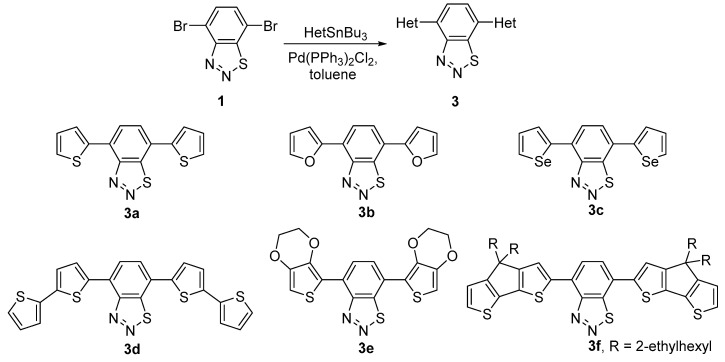
The Stille cross-coupling reactions of 4,7-dibromobenzo[*d*][1,2,3]thiadiazole **1**.

The conditions found for the synthesis of bis(thienyl) derivative **3a** were studied further (Scheme 3). In general, this protocol worked well for tributylstannanes of thiophene, furane and selenophene derivatives, and the target π–A–π compounds **3a**–**f** were obtained in moderate yields (40–61%).

**Figure 2:**
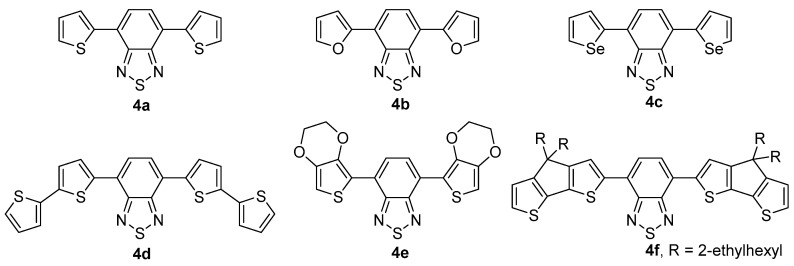
The BTD derivatives **4** obtained by earlier proposed procedures.

The π–A–π design compounds based on benzo[*c*][1,2,5]thiadiazole, also studied in this work, were synthesized from the corresponding 4,7-dibromobenzo[*c*][1,2,5]thiadiazole according to earlier proposed procedures [22,26,27,28,29,30] (Figure 2).

### 2.3. Optical Properties

**Figure 3:**
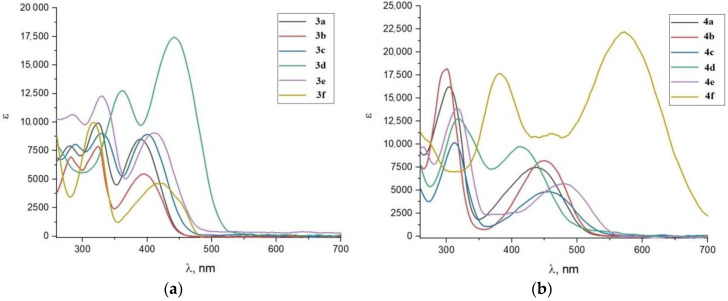
Absorption spectra of compounds **3a**–**f** (**a**) and **4a**–**f** (**b**) in DMSO solutions (5.5 × 10^−5^ M).

For understanding the effect of atom order changes in the heterocyclic part of the benzothiadiazole fragment on the optical properties of the π–A–π structures, and for comparing the regularities when changing the π-spacers in the series of compounds based on BT **4a**–**f** and isoBTD **3a**–**f**, the electronic absorption spectra of the synthesized compounds were recorded in DMSO solutions (Figure 3), and the main absorption parameters are summarized in Table 3. All dyes have two prominent absorption maxima in the visible range. The maximum in the short wavelength range (300–400 nm) corresponds to π–π* transitions of the conjugate system [31,32,33]. The intramolecular charge transfer (ICT) between the donor and acceptor parts of molecules is confirmed by the presence of a long-wave absorption maximum at 400–500 nm.

The increase in the conjugation chain with 2,2′-bithiophene and 4,4-bis(2-ethylhexyl)-4H-cyclopenta[2,1-*b*:3,4-*b*′]dithiophene (CPDT) donors accompanies the red shift of the absorption maximum at a larger wavelength for both BTD **4d**,**f** and isoBTD **3d**,**f** series compared to the dyes **4a**–**c** and **3a**–**c**. The bathochromic shift of the ICT band derivatives with CPDT donors **4f** and **3f** also indicates strong charge transfer properties among other donors.

For a number of isoBTD-based compounds, a direct correlation is observed between the shift of the ICT absorption maximum into the red region of the light spectra with an increase in the donorship of the substituents: compounds with 2,2-bithiophene **3d**, CPDT **3f** and 2,3-dihydrothieno[3,4-*b*][1,4]dioxine (EDOT) **3e** π-spacers exhibit a shift in the second absorption maxima in the region of the long wavelength compared to five-membered heterocyclic substituents **3a**–**c**. Interestingly, despite the fact that generally the BTD derivatives show similar behavior, the exception is compound **4d**, for which the short-wave maximum lies in the absorption region of the other three-membered derivatives, and the long-wave maximum undergoes a blue shift.

For compounds with five-membered heterocyclic substituents, a predictable pattern is observed: for both series of compounds based on BTD **4a**–**c** and isoBTD **3a**–**c** both absorption maxima slightly shift to the red region of the spectra during the transition from furan to selenophene substituents, which is explained by the increase in ICT due to the increase in the heteroatom radius.

Among the pairs of isomers that differ only in the order of the atoms in the acceptor cycle, there is a clear dependence in the shifts of the long-wave absorption maxima into the blue region of the spectra when passing from BTD derivatives to isoBTD. An exception to this pattern is compound **3d**, the long-wave absorption maximum of which has a pronounced red shift in comparison with its BTD **4d** analogue. The short-wave absorption maxima also follow a general pattern: compounds with the isoBTD acceptor show a bathochromic shift in comparison with their BTD analogues. The observed bathochromic shift may be related to a lower stabilization of the LUMO energy level centered on the acceptor core in isoBTD than in BTD, caused by the asymmetry of the benzothiadiazole ring. However, this case had some exceptions as well: compound **4f** has a pronounced red shift of absorption maxima. Probably, in this case, the strong donor nature of the CPDT substituents and the increased conjugation chain prevail over the contribution of the acceptor unit to the electronic structure of the molecule.

**Figure 4:**
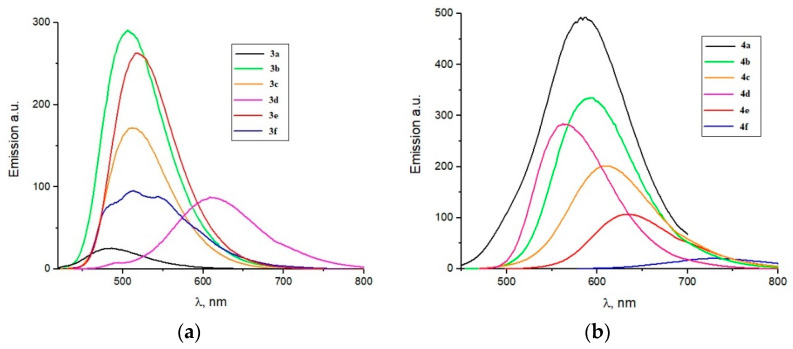
Emission spectra of compounds **3a**–**f** (**a**) and **4a**–**f** (**b**) in DMSO solutions (5 × 10^−6^ M).

**Figure 5:**
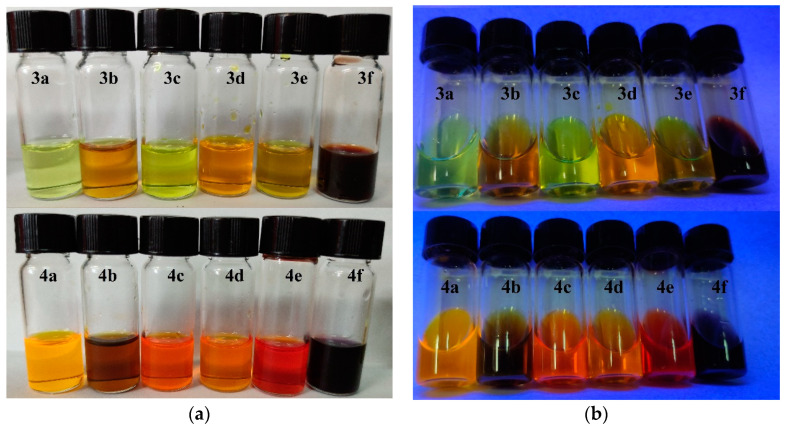
The photographs of the solutions of compounds **3a**−**f** and **4a**−**f** in DMSO (2 × 10^−3^ M) under daylight (**a**) and under UV-light (**b**).

For a deeper understanding of the influence of the molecular structure on their optical properties, we measured the luminescence spectra of the obtained compounds in DMSO solutions. The results are shown in Figure 4 and Table 3. In general, the patterns found in the analysis of the emission spectra are consistent with those described above for the ICT absorption band in UV/vis spectra. During the transition from BTD derivatives to isoBTD derivatives, a hypsochromic shift is observed for all substituents except for the 2,2′-bithiophene derivatives **3d** and **4d**. The Stokes shift for BTD derivatives is also significantly higher compared to analogous derivatives with the isoBTD acceptor, which is probably due to the more efficient ICT and a larger dipole moment of excited states of BTD derivatives [34]. The assumption that the symmetric nature of the BTD acceptor promotes intramolecular charge transfer is indirectly confirmed by the intensity of coloring of the solutions of the obtained compounds of the same concentration in daylight (Figure 5a) and under UV irradiation (Figure 5b). Another reason for the stronger red shift observed in the emission spectra may lie in more significant conformational changes in the geometry of the molecules in the excited state [35]. 2,2′-Bithiophene derivatives **3d** and **4d** demonstrate an inverse dependence again: the Stokes shift of compound **3d** with isoBTD acceptor is larger than that of compound **4d** with BTD acceptor.

Thus, the analysis of the data obtained leads to the general conclusion that the breaking of the thiadiazole ring symmetry during the transition from BTD derivatives **4** to isoBTD derivatives **3** results in an overall decrease of the luminescence intensity and a bathochromic shift in the UV/vis spectra, which may be due to a less effective intramolecular charge transfer. Nevertheless, the wavelength values of the absorption maxima in the UV/vis spectra allow us to consider compounds based on the isoBTD acceptor as promising building blocks for the creation of new materials for organic electronics.

### 2.4. Electrochemical Properties

**Figure 6:**
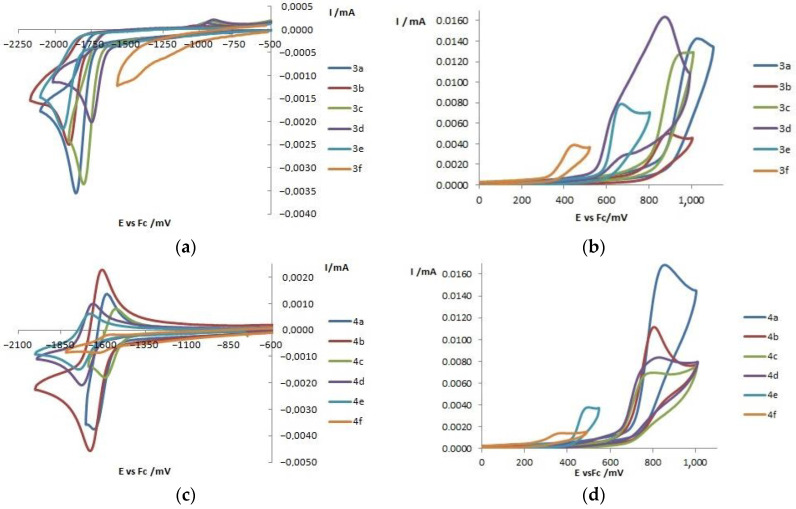
CV curves of ER (**a**,**c**) and EO (**b**,**d**) compounds in the series **3a**–**f** and **4a**–**f**, respectively, in DMF solution (0.1 M Bu_4_NBF_4_) on platinum electrode at 0.1 Vs^−1^ potential sweep rate.

To estimate the energy values of the frontier orbitals and to determine the stability of the particles formed during electron transfer, cyclic voltammetry patterns (CV curves) of BTD **4a**–**f** and isoBTD **3a**–**f** based compounds were obtained. Figure 6 shows the first-stage electrooxidation (EO) and electroreduction (ER) curves of the studied compounds, and the potential values and calculated frontier orbitals energies are summarized in Table 4. All dyes have EO and ER peaks on CV curves in DMF solution (0.1M Bu_4_NBF_4_). To calculate the energies of the lowest unoccupied molecular orbital (E_LUMO_) and the highest occupied molecular orbital (E_HOMO_), we used the peak onset values ER (E^red^_onset_) and EO (E^ox^_onset_), respectively. The values of E^red^_onset_ and E^ox^_onset_ were calculated relative to the potential of the reversible oxidation of ferrocene/ferrocenium (Fc/Fc^+^) redox pair, the absolute potential of which equals −5.1eV [36,37]. We used Equations (1) and (2) to calculate the values of E_LUMO_ and E_HOMO_:

E_HOMO_ (eV) = −|e|(E^ox^_onset, Fc/Fc+_ + 5.1), (1)

E_LUMO_ (eV) = −|e|(E^red^_onset, Fc/Fc+_ + 5.1). (2)

**Figure 7:**
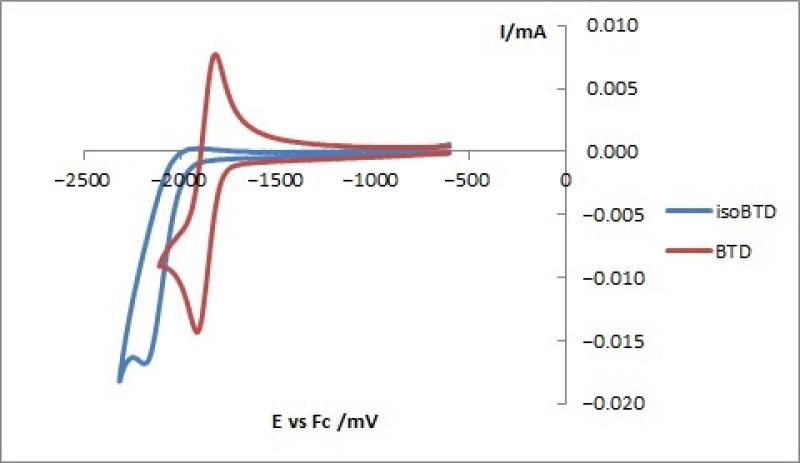
CV curves of BTD and isoBTD on a platinum electrode in DMF (0.1M Bu_4_NBF_4_) at a potential sweep rate of 0.1 Vs^−1^. The concentration of the compounds is 5.0 × 10^−3^ M.

The compounds of the BTD series **4a**–**f** are chemically reversible (Figure 6**c**), with the formation of sufficiently stable anion radicals at a low potential sweep rate of 0.1 Vs^−1^. This fact, apparently, can be considered a confirmation of electron transfer to the BTD acceptor building block in the **4a**–**f** dye series, since benzo[*c*][1,2,5]thiadiazole, which has no substituents, is also reversibly reduced (Figure 7). Unlike BTD, isoBTD recovers irreversibly and its ER potential is 200 mV more negative. That is, the E_LUMO_ energy in the case of BTD is 0.2 eV higher than for isoBTD. It should be noted that the CV curves in DMF solutions show the absence of EO peaks for both BTD and isoBTD. Since the background discharge onset potential (0.1M Bu_4_NBF_4_) is defined as approximately 1.0 V relative to Fc/Fc^+^ we can assume that the E_HOMO_ value for both compounds is significantly lower (6 eV).

The addition of substituents, depending on their electron-donating properties and their ability to delocalize the electron, shifts the ER potential of both BTD and isoBTD derivatives into the positive region of potentials in relation to the acceptor potentials, and in series **3a**–**c**,**e** and **4a**–**c**,**e** these shifts, caused by the same substituents, are almost identical in pairs. For the five-membered **3a**–**c** and **4a**–**c** heterocyclic substituents, the ER potential shift increases in the series from furan (210 mV) to selenophene (310–330 mV), which can be explained by an increase of the electron delocalization degree in the same sequence by increasing the heteroatom radius. EDOT substituent (170–180 mV) has slightly less effect on ER potentials **3e** and **4e**. As for the effect of the 2,2′-bithiophene substituent, the **3d** potential shift (360 mV) is twice that of **4d**, which may be due to the asymmetry of **3d**. The most positive ER potential turned out to be in the case of **3f**, differing from the ER potential of isoBTD by almost 1 V. Apparently, the bulky substituent CPDT in **3f** with an increased conjugation chain compared to the closely related **3d** reduces the E_LUMO_ to a minimum value of −4.10 eV among the dyes studied. No such strong effect is observed in the BTD series **4f**.

The values of EO potentials primarily determine the donor contribution of substituents to the electronic structure of dyes. EO of the studied compounds of both series is irreversible. The electrooxidation peaks for compounds **4a**–**c** with BTD have 0.15–0.20 V lower values than for compounds **3a**–**c** based on isoBTD, and in the case of EDOT substituents, the EO value of compound **4e** is much lower (by 0.40 V) than that of compound **3e**. This may be due to the more efficient conjugation in BTD systems due to the symmetry of benzo[*d*][3,2,1]thiadiazole core, and compounds with strong donor substituents appear to be most sensitive to this factor. The patterns of changes in the EO values of BTD- and isoBTD-based compounds with substitution of substituents conform to the general rules. In the series with furan (**3b**), thiophene (**3a**) and selenophene (**3c**) the oxygen-containing analogue **3b** has one of the lowest oxidation potential values, which may be due to the reduced aromatization energy [38] of the furan ring. The addition of strong donor substituents (EDOT **3e**,**4e** and CPDT **3f**,**4f**) leads to a significant decrease in the EO potential compared to compounds with five-membered heterocyclic substituents (**3a**–**c**,**4a**–**c**), both in the series BTD and in the series isoBTD derivatives. Interestingly, compound **3d** with the 2,2′-bithiophene substituent also has a reduced EO potential compared to the **3a**–**c**, derivatives, which is consistent with the increased donor strength of this substituent and the extended five-membered system conjugation chain in **3d**. Nevertheless, for compound **4d** the EO potential value is at the same level as that for compound **4a**.

The electrochemical band gap (E_g_) values were also calculated by means of the obtained electrochemical parameters. In the series **3a**–**c** the value of E_g_ decreases in the following order: **3a** (2.59 eV), **3b** (2.51 eV), **3c** (2.48 eV). For the **4a**–**c** series, the E_g_ values are 2.25, 2.08 and 2.09 eV, respectively, which is generally lower than in the **3a**–**c** series. It is interesting to note the opposite effect of the EDOT and 2,2′-bithiophene substituents on the EO potentials of the compounds **3d,e** and **4d,e** and, consequently, on the E_g_ value: for the compound **3d** (2.18 eV) < **4d**(2.25 eV), and for **3e** (2.41 eV) > **4e** (2.02 eV). But the most different from all the compounds studied is **3f**, which has an E_g_ value of 1.36 eV, due to its low EO potential (0.36 V) and the most positive ER potential of all obtained values (−1.0 V). The dye **4f**, which oxidizes at the lowest potential (0.25 V), has a value of E_g_ = 1.76 eV.

In general, the value of the band gap decreases with increasing donor strength of the substituents: the minimum values are observed for compounds with CPDT substituents **3f** and **4f**. During the transition from BTD derivatives to isomeric compounds with the isoBTD acceptor, an increase in E_g_ is observed for all pairs of synthesized compounds except for the 2,2′-bithiophene derivatives **3d** and **4d**.

Thus, the electrochemical characteristics obtained for the produced **3a**–**f** series and their comparison with **4a**–**f** allow us to state that isoBTD-based dyes can have a wide spectrum of properties, enabling their application in various fields of organic electronics.

## 3. Experimental Section

### 3.1. Materials and Reagents

The chemicals were purchased from the commercial sources (Sigma-Aldrich, St. Louis, MO, USA) and used as received. Benzo[*d*][1,2,3]thiadiazole [39], thiophen-2-ylboronic acid [40], 4,4,5,5-tetramethyl-2- (thiophen-2-yl)-1,3,2-dioxaborolane [41], tributyl(thiophen-2-yl)stannane [42], tributyl(furan-2-yl) stannane [42], tributyl(selenophen-2-yl)stannane [42], [2,2′-bithiophen]-5-yltri-butylstannane [42], tributyl(2,3-dihydrothieno[3,4-*b*][1,4]dioxin-5-yl)stannane [42], (4,4-bis(2-ethylhexyl)-4*H*-cyclopenta [2,1-*b*:3,4-*b*′]dithiophen-2-yl)tributylstannane [42], 4,7-dibromobenzo[*c*][1,2,5]thiadiazole [43], 4,7-di(thiophen-2-yl)benzo[*c*][1,2,5]thiadiazole (**4a**) [22], 4,7-di(furan-2-yl)benzo[*c*][1,2,5]thiadiazole (**4b**) [26], 4,7-di(selenophene-2-yl)benzo[*c*][1,2,5]thiadiazole (**4c**) [27], 4,7-di([2,2′-bithiophen]-5-yl)benzo[*c*][1,2,5]thiadiazole (**4d**) [28], 4,7-bis(2,3-dihydrothieno[3,4-*b*][1,4]dioxin-5-yl)benzo[*c*][1,2,5]thiadiazole (**4e**) [29], 4,7-bis(4,4-bis(2-ethylhexyl)-4*H*-cyclopenta[2,1-*b*:3,4-*b*′-]dithiophen-2-yl)benzo[*c*][1,2,5]thiadiazole (**4f**) [30] were prepared according to the previously described protocols. All synthetic operations were performed under a dry argon atmosphere. The solvents were purified by distillation over the appropriate drying agents.

### 3.2. Analytical Instruments

The solution UV-visible absorption spectra were recorded using an OKB Spektr SF-2000 UV/vis/NIR spectrophotometer (Saint-Petersburg, Russia) controlled with SF-2000 software. All samples were measured in a 1 cm quartz cell at room temperature with a 3.7 × 10^−5^ mol/mL concentration in DMSO. The luminescence spectra were recorded on the Agilent Cary Eclipse instrument (Santa Clara, CA, USA). The melting points were determined on a Kofler hot-stage apparatus and were uncorrected. ^1^H and ^13^C NMR spectra were taken with a Bruker AM-300 machine (Bruker Ltd., Moscow, Russia) with TMS as the standard. *J* values are given in Hz. MS spectra (EI, 70 eV) were obtained with a Finnigan MAT INCOS 50 instrument (Thermo Finnigan LLC, San Jose, CA, USA). High-resolution MS spectra were measured on a Bruker micrOTOF II instrument using electrospray ionization (ESI). The measurement was operated in a positive ion mode (interface capillary voltage −4500 V) or in a negative ion mode (3200 V); the mass range was from *m*/*z* 50 to *m*/*z* 3000 Da; external or internal calibration was performed with Electrospray Calibrant Solution (Fluka Chemicals Ltd., Gillingham, UK). A syringe injection was used for solutions in acetonitrile, methanol, or water (flow rate 3 μL·min^−1^). Nitrogen was applied as a dry gas; the interface temperature was set at 180 °C. IR spectra were measured with a Bruker “Alpha-T” instrument (Bruker, Billerica, MA, USA) in KBr pellets. Electrochemical measurements were carried out in a dry argon atmosphere using an IPC Pro MF potentiostat (Econix, Moscow, Russia). The redox properties of compounds were determined using cyclic voltammetry in a three-electrode electrochemical system. A three-electrode system consisting of platinum as the working electrode with an area of 0.8 mm^2^, platinum wire as the counter electrode, and a saturated calomel electrode (SCE) as the reference electrode was employed. The reduction and oxidation potentials were determined in DMF, using 0.1 mol L^−1^ *n*-Bu_4_NBF_4_ as the supporting electrolyte. The cyclic voltammetry (CV) measurements were performed with the use of scan rates of 0.1–5.0 V s^−1^. The first reduction/oxidation potentials were referenced to the internal standard redox couple Fc/Fc^+^. Ferrocene was added to each sample solution at the end of the experiment and employed for calibration.

### 3.3. Computational Details

DFT calculations were performed with the Gaussian 16 Rev C.01. M11 DFT functional with 6-31+g(d) basis set was used for all calculations. The calculations were performed in dichloromethane (PCM model). Data from various X-ray diffraction experiments were used as starting points for geometry optimizations. Cartesian coordinates are given in angstroms; absolute energies for all substances are given in Hartree units. Analysis of vibrational frequencies was performed for all optimized structures. All compounds were characterized by only real vibrational frequencies. Wavefunction stability, with *stable* as the keyword, was also checked for each molecule.

### 3.4. Synthesis of 4,7-Dibromobenzo[*d*][1,2,3]thiadiazole ***1***

In a 100 mL round-bottom flask benzo[*d*][1,2,3]thiadiazole **2** (500 mg, 3.63 mmol) was dissolved in concentrated sulfuric acid (95%, 26 mL) and (2.58 g, 16.7 mmol) NBS was added to the resulting solution. The mixture was heated to 50 °C and maintained in these conditions for 6 h. Then the reaction mixture was cooled to room temperature and was poured onto ice water (200 mL). After that, the mixture was extracted with chloroform (150 mL), the organic layer was separated and washed with water (5 × 70 mL), then dried over sodium sulfate and concentrated in vacuo to give 848 mg (80%) of light-brown solid, mp 148–149 °C (lit. [9] 149–151 °C). The ^1^H, ^13^C NMR, and HRMS spectra correspond to the literature data [9].

### 3.5. General Procedure for the Synthesis of Compounds ***3***

In a 50 mL round-bottom flask, 4,7-dibromobenzo[*d*][1,2,3]thiadiazole **1** (100 mg, 0.34 mmol) and heterocyclic tributylstannane (0.75 mmol) were dissolved in toluene (20 mL). The mixture was degassed for 20 min with a stream of argon, and PdCl_2_(PPh_3_)_2_ (10 mg, 0.009 mmol) was added. After refluxing for 6 h, the reaction mixture was diluted with EtOAc (20 mL) and plugged through a thin pad of Celite. Then the filtrate was evaporated. The precipitate was purified by column chromatography (Silica gel Merck 60, petroleum ether, and then petroleum ether–ethyl acetate mixtures 200:1 to 50:1) (See Supplementary Materials).

*4,7-Di(thiophen-2-yl)benzo[*d*][1,2,3]thiadiazole* (**3a**). Yield 61 mg (60%). Orange solid, mp 118–119 °C. ^1^H NMR (300 MHz, CDCl_3_, δ, ppm): 8.12 (d, *J* = 3.8 Hz, 1H), 7.84–7.74 (m, 2H), 7.43 (d, 1H, *J* = 5.1), 7.40–7.33 (m, 2H), 7.19–7.09 (m, 2H). ^13^C NMR (75 MHz, CDCl_3_, δ, ppm): 155.2, 142.1, 140.3, 139.0, 129.6, 128.8, 128.5, 128.3, 128.0, 127.08, 126.5, 125.6, 125.5, 125.4. HRMS-ESI (*m*/*z*): calcd for (C_14_H_9_N_2_S_3_) [M + H]^+^ 300.9925, found 300.9922. MS (EI, 70 eV), *m*/*z* (I, %):300 (80), 272 (100), 240 (17), 227 (43), 214 (7), 195 (17), 69 (15), 57 (14), 45 (28). UV-Vis (CH_2_Cl_2_, λ_max_, nm/logε): 324/4.00, 390/3.93. IR, ν, cm^−1^: 3444, 2925, 2856, 1627, 1467, 1272, 1015, 821, 698. R_f_ = 0.35 (petroleum ether/ethyl acetate—10/1).

*4,7-Di(furan-2-yl)benzo[*d*][1,2,3]thiadiazole* (**3b**). Yield 56 mg (61%). Orange solid, mp 134–136 °C. ^1^H NMR (300 MHz, CDCl_3_, δ, ppm): 8.06 (d, *J* = 7.9, 1H), 7.95 (d, *J* = 8.1, 1H), 7.88 (d, 1H, *J* = 3.4), 7.64 (m, 2H), 6.85 (d, 1H, *J* = 3.4), 6.63 (d, 2H, *J* = 14.0). ^13^C NMR (75 MHz, CDCl_3_, δ, ppm): 153.5, 150.9, 149.5, 143.2, 143.1, 136.6, 124.8, 123.6, 122.3, 121.1, 113.5, 112.5, 112.2, 107.5. HRMS-ESI (*m*/*z*): calcd for (C_14_H_9_N_2_O_2_S) [M + H]^+^ 269.0379, found 269.0379. MS (EI, 70eV), *m*/*z* (I, %): 268 (100), 240 (36), 211 (36), 184 (28), 152 (10), 139 (40), 92 (10), 69 (10), 39 (10), 29 (15), 18 (26). UV-Vis (CH_2_Cl_2_, λ_max_, nm/logε): 324/3.89, 394/3.74. IR, ν, cm^−1^: 3435, 3119, 2962, 1591, 1503, 1341, 1281, 1080, 1015, 822, 730. R_f_ = 0.33 (petroleum ether/ethyl acetate—10/1).

*4,7-Di(selenophen-2-yl)benzo[*d*][1,2,3]thiadiazole* (**3c**). Yield 57 mg (42%). Orange solid, mp 123–124 °C. ^1^H NMR (300 MHz, CDCl_3_, δ, ppm): 8.29–8.21 (m, 2H), 8.14 (d, 1H, *J* = 5.6), 7.94 (d, 1H, *J* = 7.9), 7.81 (d, 1H, *J* = 7.9), 7.65 (d, 1H, *J* = 3.8), 7.51–7.42 (m, 2H). ^13^C NMR (75 MHz, CDCl_3_, δ, ppm): 155.0, 148.2, 143.7, 140.4, 135.2, 133.07, 132.3, 131.5, 131.0, 130.4, 129.6, 127.9, 127.7, 124.8. HRMS-ESI (*m*/*z*): calcd for (C_14_H_9_N_2_SSe_2_) [M + H]^+^ 396.8816, found 396.8813. MS (EI, 70eV), *m*/*z* (I, %): 396 (78), 368 (54), 288 (100), 208 (58), 195 (19), 182 (19), 163 (19), 117 (22), 93 (70), 81 (21), 69 (22), 39 (34). UV-Vis (CH_2_Cl_2_, λ_max_, nm/logε): 328/3.95, 402/3.95. IR, ν, cm^−1^: 3434, 2926, 2359, 1636, 1471, 1273, 1217, 828, 684. R_f_ = 0.28 (petroleum ether/ethyl acetate—10/1).

*4,7-Di([2,2′-bithiophen]-5-yl)benzo[*d*][1,2,3]thiadiazole* (**3d**). Yield 63 mg (40%). Orange solid, mp 146–148 °C. ^1^H NMR (300 MHz, CDCl_3_, δ, ppm): 8.10 (d, 1H, *J* = 3.9,), 7.90–7.79 (m, 2H), 7.36 (d, 1H, *J* = 3.9), 7.30 (d, 1H, *J* = 3.5), 7.28–7.21 (m, 5H), 7.08–7.01 (m, 2H). ^13^C NMR (75 MHz, CDCl_3_, δ, ppm): 156.7, 154.9, 148.1, 143.6, 140.3, 135.1, 133.0, 132.2, 131.5, 130.9, 130.3, 129.5, 127.9, 127.8, 127.6, 124.7. HRMS-ESI (*m*/*z*): calcd for (C_22_H_13_N_2_S_5_) [M + H]^+^ 464.9636, found 464.9637. MS (EI, 70eV), *m*/*z* (I, %): 464 (100), 436 (92), 402 (19), 309 (17), 149 (21), 127 (31), 108 (12), 69 (27), 45 (46), 18 (38). UV-Vis (CH_2_Cl_2_, λ_max_, nm/logε): 362/4.11, 442/4.24. IR, ν, cm^−1^: 3434, 3063, 2959, 2928, 1727, 1480, 1271, 1072, 837, 792, 666. R_f_ = 0.24 (petroleum ether/ethyl acetate—10/1).

*4,7-Bis(2,3-dihydrothieno[3,4-b][1,4]dioxin-5-yl)benzo[*d*][1,2,3]thiadiazole* (**3e**). Yield 69 mg (49%). Orange solid, mp 184–186 °C. ^1^H NMR (300 MHz, CDCl_3_, δ, ppm): 8.39 (d, 1H, *J* = 8.1), 8.09 (d, 1H, *J* = 8.1), 6.61 (s, 1H), 6.51 (s, 1H), 4.43–4.31 (m, 8H). ^13^C NMR (75 MHz, CDCl_3_, δ, ppm): 142.5, 141.7, 140.9, 139.9, 139.3, 131.0, 127.9, 125.7, 123.1, 115.6, 113.4, 103.4, 99.9, 99.8, 65.2, 65.0, 64.6, 64.5. HRMS-ESI (*m*/*z*): calcd for (C_18_H_13_N_2_O_4_S_3_) [M + H]^+^ 417.0031, found 417.0032. MS (EI, 70eV), *m*/*z* (I, %): 416 (100), 357 (36), 329 (18), 291 (51), 207 (24), 175 (22), 93 (31), 69 (25), 45 (41), 27 (40). UV-Vis (CH_2_Cl_2_, λ_max_, nm/logε): 330/4.09, 410/3.96. IR, ν, cm^−1^: 3435, 2926, 2855, 2339, 1728, 1502, 1479, 1073, 801. R_f_ = 0.26 (petroleum ether/ethyl acetate—5/1).

*4,7-Bis(4,4-bis(2-ethylhexyl)-4H-cyclopenta[2,1-b:3,4-b′]dithiophen-2-yl)benzo[*d*][1,2,3]thiadiazole* (**3f**). Yield 134 mg (42%). Red-purple oil, ^1^H NMR (300 MHz, CDCl_3_, δ, ppm): 8.21 (d, 1H, *J =* 4.4), 7.90 (d, 1H, *J =* 7.8), 7.83 (d, 1H, *J =* 7.8), 7.32 (d, 1H, *J =* 1.9), 7.27–7.26 (m, 1H), 7.22 (d, 1H, *J =* 4.8), 7.01–6.95 (m, 2H), 2.04–1.87 (m, 8H), 1.05–0.87 (m, 33H), 0.76 (m, 10H), 0.68–0.57 (m, 17H). ^13^C NMR (75 MHz, CDCl_3_, δ, ppm): 158.8, 158.5, 158.2, 154.7, 154.7, 139.9, 138.0, 136.8, 136.4, 131.9, 129.9, 129.9, 126.1, 125.9, 125.6, 125.4, 125.4, 124.2, 124.1, 123.9, 122.4, 122.4, 120.4, 120.3, 53.9, 53.9, 53.8, 43.2, 43.2, 43.1, 35.1, 35.1, 34.2, 34.2, 34.2, 34.2, 34.1, 28.7, 28.6, 28.6, 28.5, 27.4, 27.4, 27.3, 22.7, 22.7, 22.7, 14.0, 14.0, 13.9, 13.9, 10.8, 10.7, 10.7, 10.6, 10.6. HRMS-ESI (*m*/*z*): calcd for (C_56_H_76_N_2_S_5_) [M]^+^ 936.4606, found 936.4607. MS (EI, 70eV), *m*/*z* (I, %): 934 (1), 661 (8), 647 (8), 316 (13), 191 (22), 147 (18), 111 (22), 99 (35), 86 (37), 75 (72), 57 (100), 42 (98), 29 (46). UV-Vis (CH_2_Cl_2_, λ_max_, nm/logε): 316/4.00, 422/3.67. IR, ν, cm^−1^: 3402, 2957, 2924, 2854, 1672, 1460, 1430, 1081, 801. R_f_ = 0.69 (petroleum ether/ethyl acetate—10/1).

## 4. Conclusions

In the present article we report the synthesis of 4,7-dibromobenzo[*d*][1,2,3]thiadiazole 1 and the π–A–π building blocks based on it and study their physicochemical and spectral properties. As a result, an efficient method for the synthesis of 4,7-dibromobenzo[*d*][1,2,3]thiadiazole 1 has been developed. The study of different variants of the Suzuki, Stille, and the direct C-H heteroarylation cross-coupling reactions showed that the most stable results in obtaining π–A–π-type structures based on the isoBTD internal acceptor were achieved in the Stille reaction. Comparison of isoBTD with its isomer BTD showed that, according to quantum mechanical calculations and cyclic voltammetry, it has a slightly higher value of E_LUMO_, and the value of the band gap for isoBTD is the highest among all studied electron-deficient heterocycles. In the UV/vis spectra of the series of π–A–π compounds, a red shift of the absorption maxima is predominantly observed in the transition from compounds based on the BTD acceptor to compounds with isoBTD, and in almost all cases a lower absorption capacity is observed for compounds of isoBTD type as well as a less intense luminescence in the corresponding spectra. However, the addition of the 2,2′-bithiophene fragment as a π-spacer led to an unexpected increase of the extinction coefficient in the UV/vis spectra along with a blue shift of both absorption maxima for 4,7-di([2,2′-bithiophen]-5-yl)benzo[*d*][1,2,3]thiadiazole 3d compared to 4,7-di([2,2′-bithiophen]-5-yl)benzo[*c*][1,2,5]thiadiazole 4d. Thus, we found that the electron acceptor unit 4,7-dibromobenzo[*d*][1,2,3]thiadiazole 1, which is easily obtained in two steps with high yields from commercially available reagents, can exhibit promising properties in an appropriate design of LED and solar cell components.

## Figures and Tables

**Table 1:** Optimization condition of bromination reaction of benzo[*d*][1,2,3]thiadiazole **2**.

Entry	2:NBS	Acid	Conc. of 2 in Acid	Conditions	Yield of 1 (%)
1	1:2.2	H_2_SO_4_	0.07 M	50 °C, 16 h	81
2	1:4	H_2_SO_4_	0.07 M	50 °C, 6 h	52
3	1:4	H_2_SO_4_	0.14 M	50 °C, 6 h	83
4	1:4	H_2_SO_4_	0.28 M	50 °C, 12 h	33
5	1:8	H_2_SO_4_	0.14 M	50 °C, 6 h	0
6	1:2.2	-	-	CCl_4_, Δ, 12 h	0
7	1:2.2	TfOH	1.14 M	CHCl_3_, Δ, 12 h	0
8	1:2.2	CF_3_CO_2_H	0.28 M	60 °C, 6 h	0
9	1:2.2	TfOH	0.28 M	50 °C, 6 h	84
10 ^a^	1:4	H_2_SO_4_	0.14 M	50 °C, 6 h	80

^a^ This experiment was performed on a big scale (for 0.1 mol of **2**).

**Table 2:** Cross-coupling of 4,7-dibromobenzo[*d*][1,2,3]thiadiazole **1** with thiophene derivatives.

Entry	X (Equiv.)	Catalyst	Solvent	Base	Temp. (°C)	Time (h)	Yield of 1 (%)
Suzuki-Miyaura coupling							
1	B(OH)_2_ (2.2)	Pd(PPh_3_)_2_	THF/H_2_O	K_2_CO_3_	80	6	48
2	B(OH)_2_ (2.2)	Pd(dppf)Cl_2_	H_2_O	Et_3_N	90	7	5
3	B(OH)_2_ (2.2)	Pd(PPh_3_)_4_	toluene	K_2_CO_3_	90	7	9
4	B(OH)_2_ (2.2)	Pd(PPh_3_)_4_	ethanol/ benzene	Na_2_CO_3_	80	7	54
5	B(OH)_2_ (2.2)	Pd(PPh_3_)_4_	dioxane/ H_2_O	K_2_CO_3_	90	7	50
6	BPin (2.2)	Pd(PPh_3_)_4_	THF/H_2_O	K_2_CO_3_	80	6	43
7	BPin (2.2)	Pd(PPh_3_)_4_	H_2_O/toluene	K_2_CO_3_	90	8	36
Stille coupling							
8	SnBu_3_ (2.2)	Pd(PPh_3_)_2_Cl_2_	toluene	-	110	10	60
9	SnBu_3_ (2.2)	Pd(PPh_3_)_2_Cl_2_	THF	-	66	10	52
10	SnBu_3_ (2.2)	Pd(PPh_3_)_2_Cl_2_	dioxane	-	100	10	45
11	SnBu_3_ (2.2)	Pd(PPh_3_)_4_	DMF, toluene	-	100	8	54
12	SnBu_3_ (2.2)	Pd(PPh_3_)_4_	toluene	-	90	8	59
13	SnBu_3_ (2.2)	Pd_2_dba_3_, (*t*-Bu)_3_P	THF	-	85	8	60
Direct C-H arylation							
14	H (10)	Pd(OAc)_2_	DMA	AcOK	155	3	24
15	H (10)	Pd(OAc)_2_, CsF, TBAB	DMA	PivOCs	80	1	33
16	H (10)	Pd(OAc)_2_	DMF	PivOK	80	4	12

**Table 3:** Photophysical properties of synyhesized compounds **3a**–**f** and **4a**–**f** in DMSO solutions (5.5 × 10^−5^ M).

Dye	λ_max1_, nm	ε_max1_, M^−1^ cm^−1^	λ_max2_, nm	ε_max2_, M^−1^ cm^−1^	λ_emis_, nm	Stoke’s Shift, nm
**3a**	329	9923	389	8499	486	97
**3b**	334	7867	400	5455	506	106
**3c**	334	8995	413	8916	512	99
**3d**	367	12,756	455	17,425	612	153
**3e**	334	12,282	422	9046	516	94
**3f**	320	9973	434	4643	544	110
**4a**	311	16,228	434	7461	586	152
**4b**	302	18,190	442	8209	594	152
**4c**	320	10,168	461	4824	612	151
**4d**	323	12,774	430	9731	562	132
**4e**	325	13,877	488	5710	632	144
**4f**	383	17,677	569	22,211	724	155

**Table 4:** Electrochemical properties of the BTD, **4a**–**f**, isoBTD, and **3a**–**f** in DMF solutions.

Dye	E^red^_onset_ (vs. Fc/Fc^+^) ^a^, V	E^ox^_onset_ (vs. Fc/Fc^+^) ^a^, V	E_LUMO_^b^, eV	E_HOMO_^b^, eV	E_g_^c^, eV
**isoBT** **D**	−1.99	-	−3.11	-	-
**3a**	−1.72	0.87	−3.38	−5.97	2.59
**3b**	−1.78	0.73	−3.32	−5.83	2.51
**3c**	−1.68	0.80	−3.42	−5.90	2.48
**3d**	−1.63	0.55	−3.47	−5.65	2.18
**3e**	−1.82	0.59	−3.28	−5.69	2.41
**3f**	−1.00	0.36	−4.10	−5.46	1.36
**BT** **D**	−1.79	-	−3.31	-	-
**4a**	−1.53	0.72	−3.57	−5.82	2.25
**4b**	−1.56	0.52	−3.54	−5.62	2.08
**4c**	−1.46	0.63	−3.64	−5.73	2.09
**4d**	−1.61	0.64	−3.49	−5.74	2.25
**4e**	−1.61	0.41	−3.49	−5.51	2.02
**4f**	−1.51	0.25	−3.59	−5.35	1.76

^a^ Here E^ox^_onset_ and E^red^_onset_ are a linear extrapolation of the low reduction potential side of the first oxidation or reduction wave respectively to the base line relative to Fc/Fc^+^ respectively. ^b^ Energies of frontier orbitals were calculated according to Equations (1) and (2). ^c^ E_g_ = E_LUMO_ − E_HOMO_.

## Data Availability

Not applicable.

## Supplementary Materials

The following are available online: ^1^H and ^13^C NMR spectra for compounds **3a**–**3f** and DFT calculations data.

## Sample Availability

Samples of the compounds **3a**–**3f** are available from the authors.
