# Supplementary material for: Benzothiadiazole vs. iso-Benzothiadiazole: Synthesis, Electrochemical and Optical Properties of D–A–D Conjugated Molecules Based on Them
Source: Molecules. 2021 Aug 14;26(16):4931. doi: 10.3390/molecules26164931 (PMC8399333; doi:10.3390/molecules26164931)

Supporting Information

Benzothiadiazole *vs.* *iso*-benzothiadiazole: if the order of the addents changes, the sum does not stay the same

Nikita S. Gudim [1], Ekaterina A. Knyazeva [1,2], Ludmila V. Mihalchenko [1], Ivan S. Golovanov [1], Vadim V. Popov [2], Natalia V. Obruchnikova [1], and Oleg A. Rakitin [1,2,]*

[1] N. D. Zelinsky Institute of Organic Chemistry, Russian Academy of Sciences, 119991 Moscow, Russian Federation; orakitin@ioc.ac.ru (O.A.R.); nikitosgudim@gmail.com (N.S.G.); katerina_knyazev@ioc.ac.ru (E.A.K.)
[2] Nanotechnology Education and Research Center, South Ural State University, 454080 Chelyabinsk, Russia; rakitinoa@susu.ru (O.A.R.); katerina_knyazev@mail.ru (E.A.K.)
* Correspondence: orakitin@ioc.ac.ru; Tel.: +7 499 135 5327

Table of Contents

1. **^{1}H and ^{13}C NMR spectra** **S2**
2. **DFT calculations** **S3**

1. ^{1}H and ^{13}C NMR spectra

4,7-di(thiophen-2-yl)benzo[d][1,2,3]thiadiazole (3a)

^{1}H NMR (300 MHz)

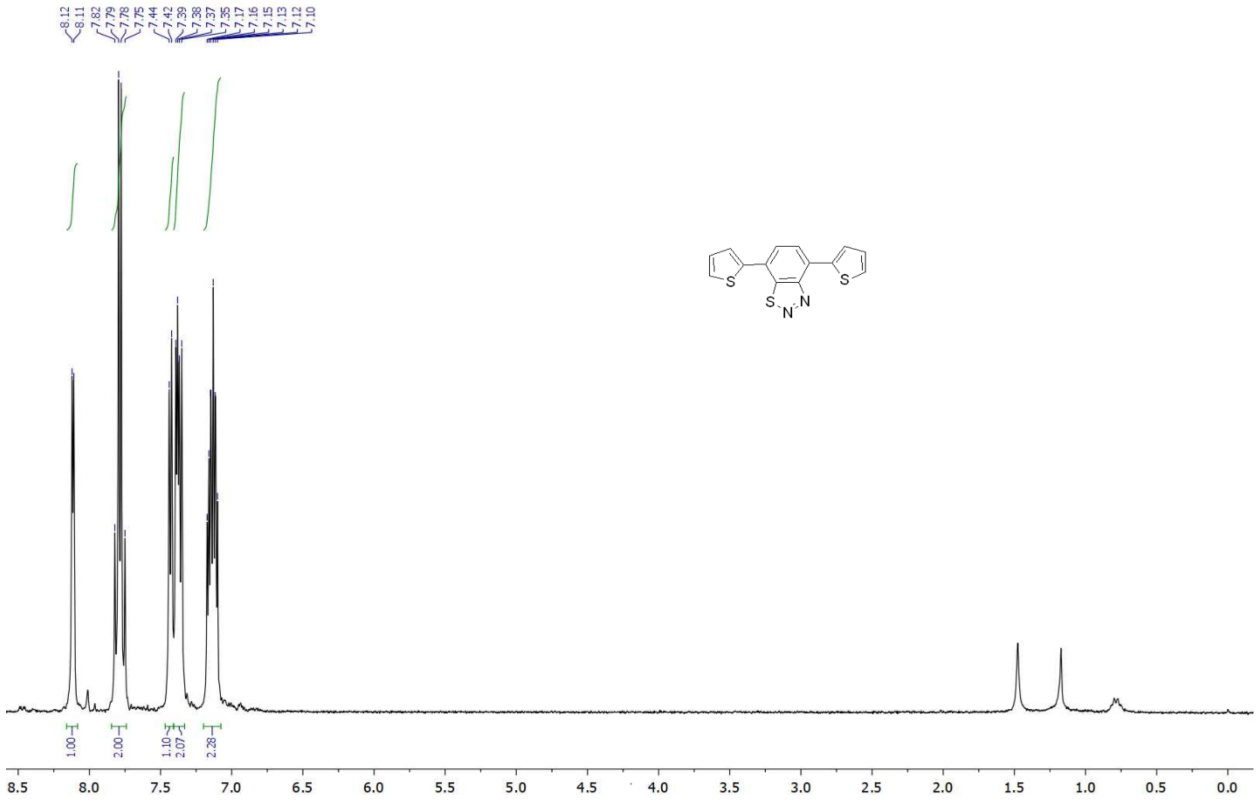

^{13}C NMR (75 MHz)

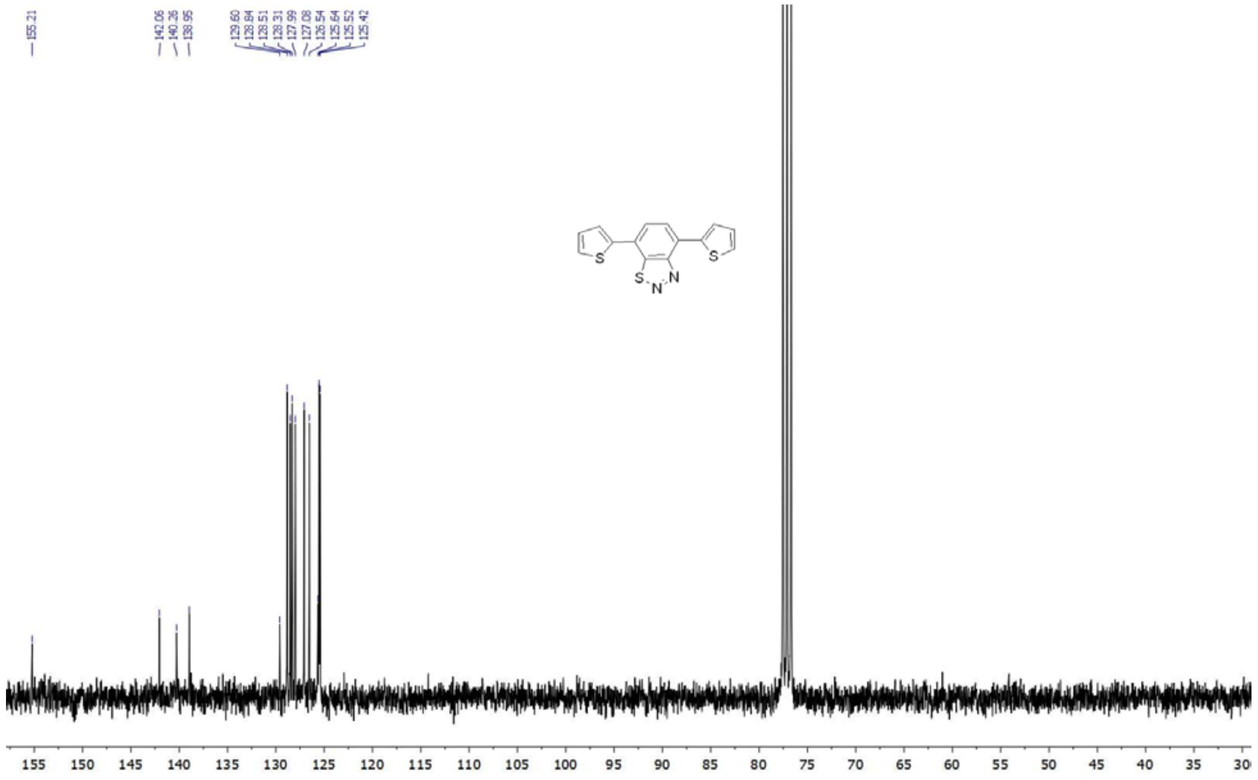

4,7-di(furan-2-yl)benzo[d][1,2,3]thiadiazole (3b)

^{1}H NMR (300 MHz)

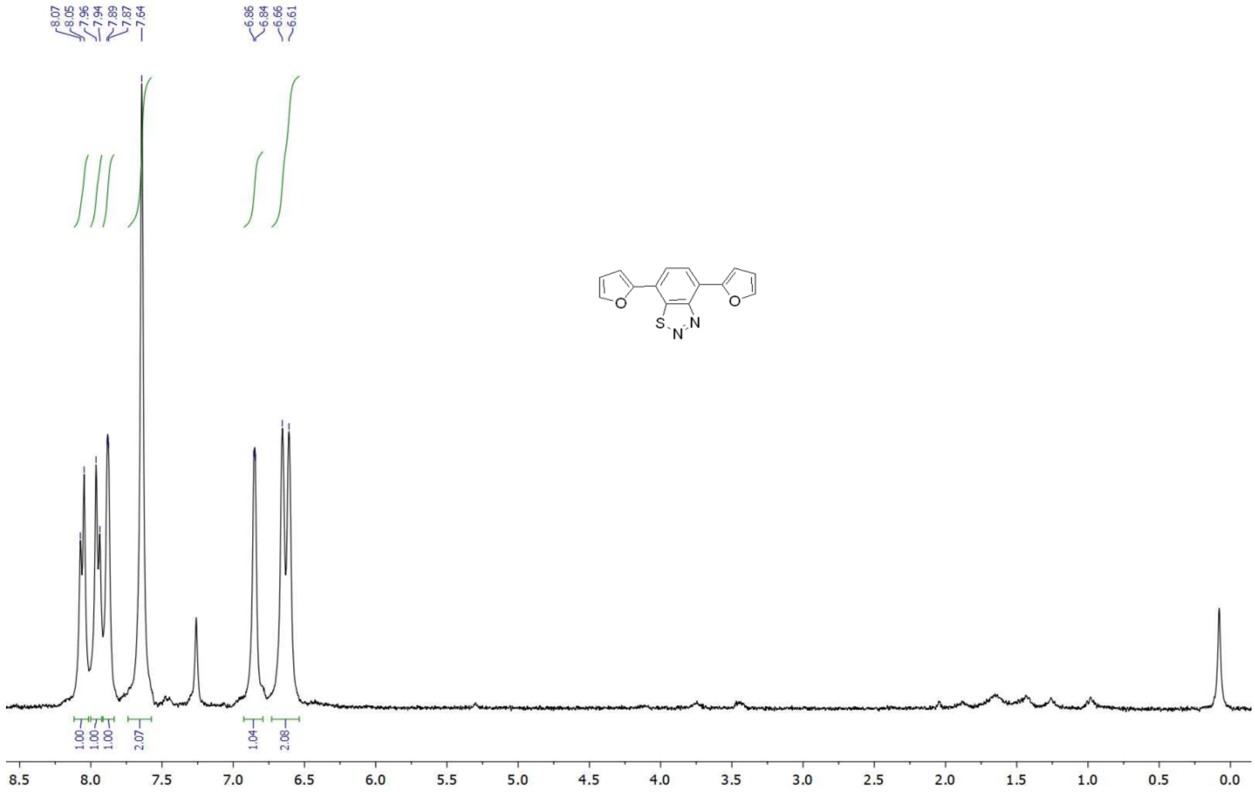

^{13}C NMR (75 MHz)

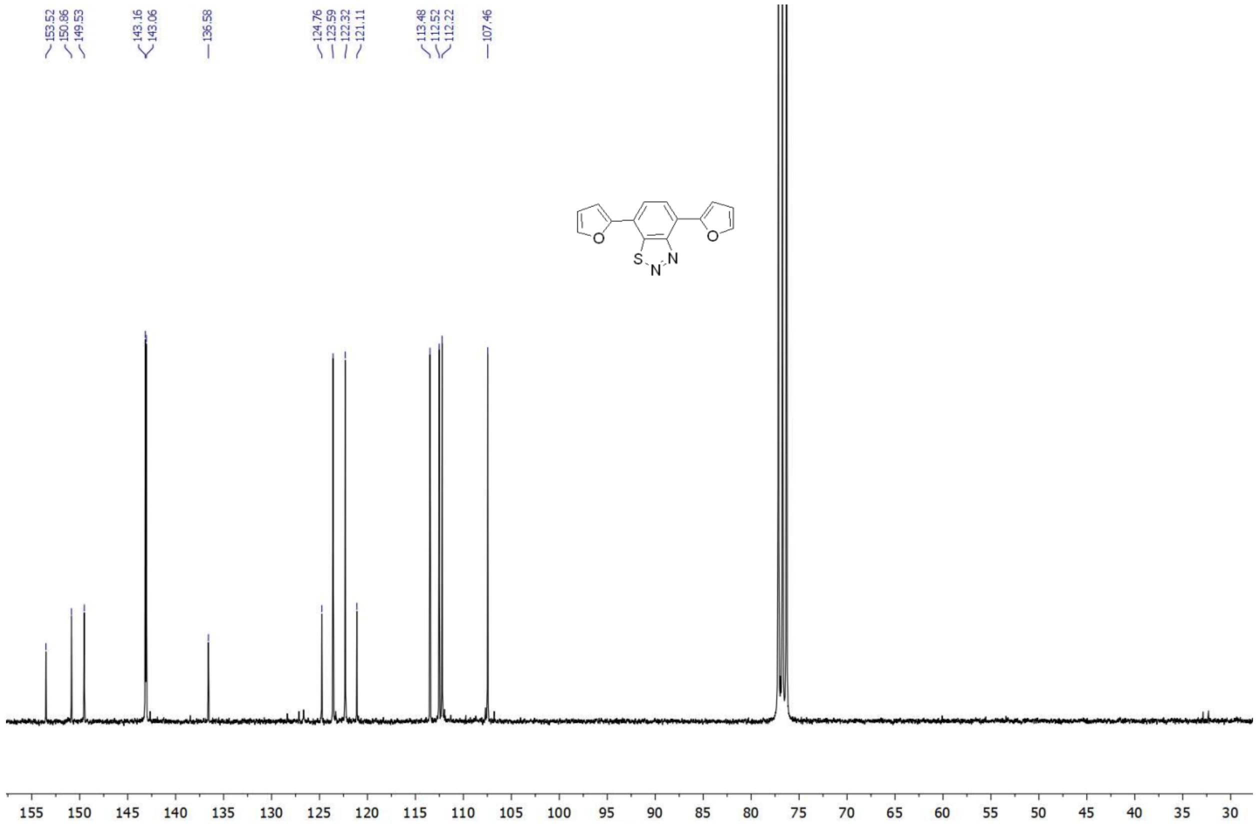

4,7-di(selenophen-2-yl)benzo[d][1,2,3]thiadiazole (3c)

^{1}H NMR (300 MHz)

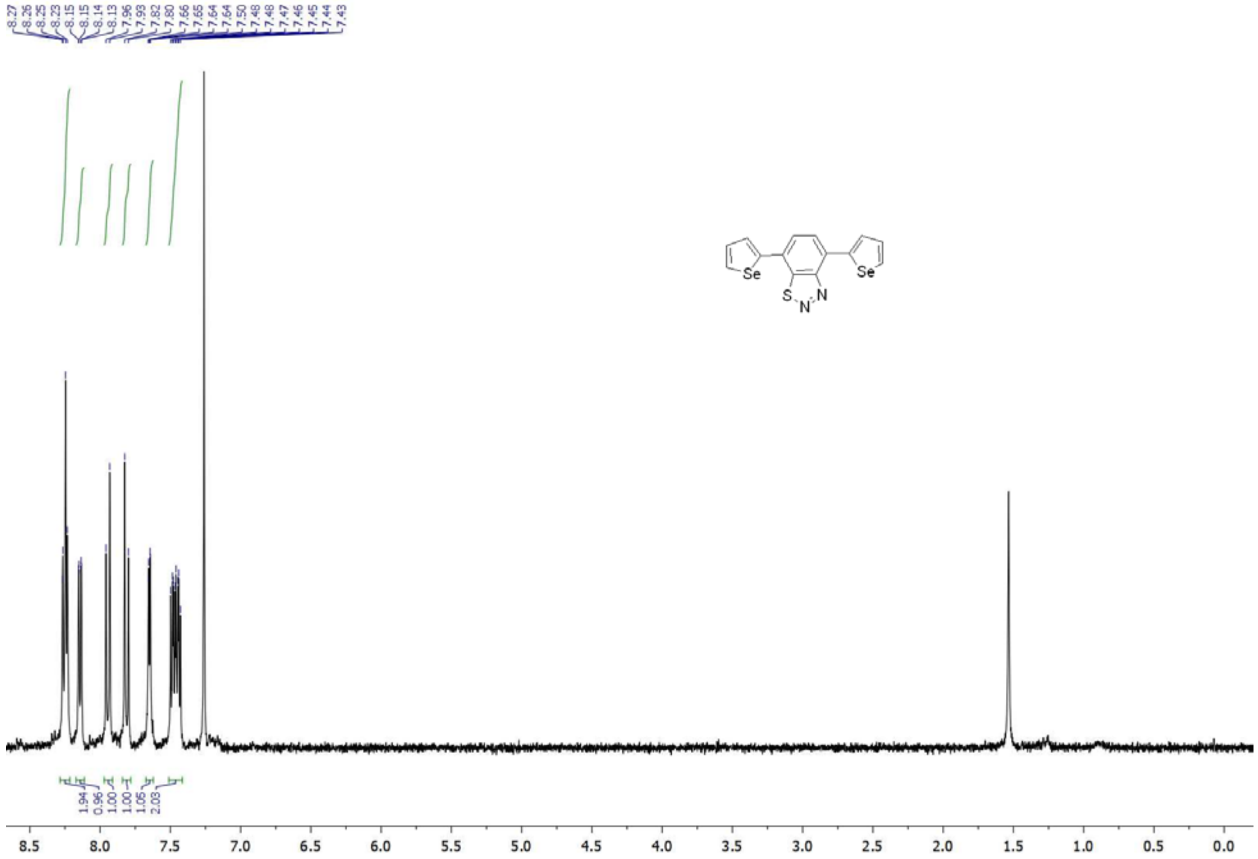

^{13}C NMR (75 MHz)

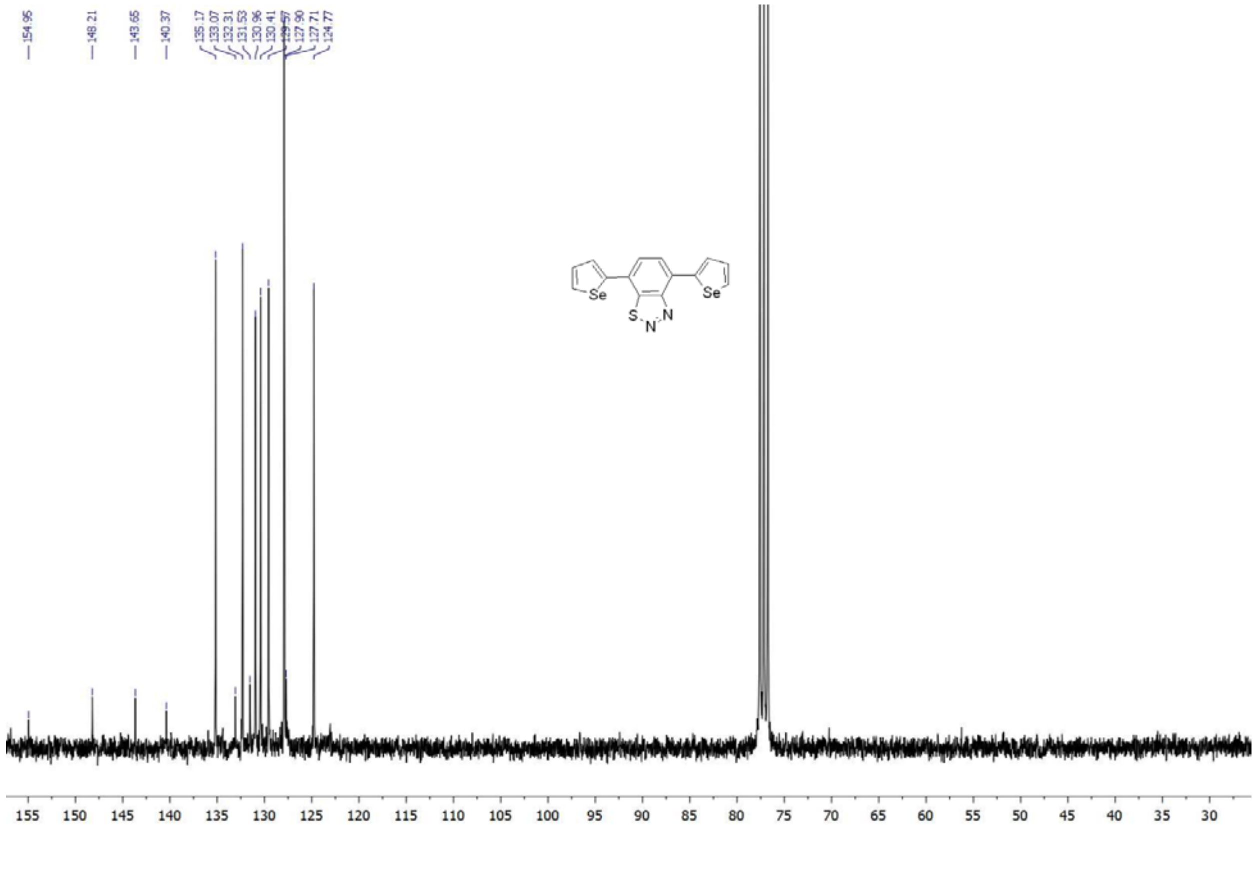

4,7-di([2,2'-bithiophen]-5-yl)benzo[d][1,2,3]thiadiazole (3d)

^{1}H NMR (300 MHz)

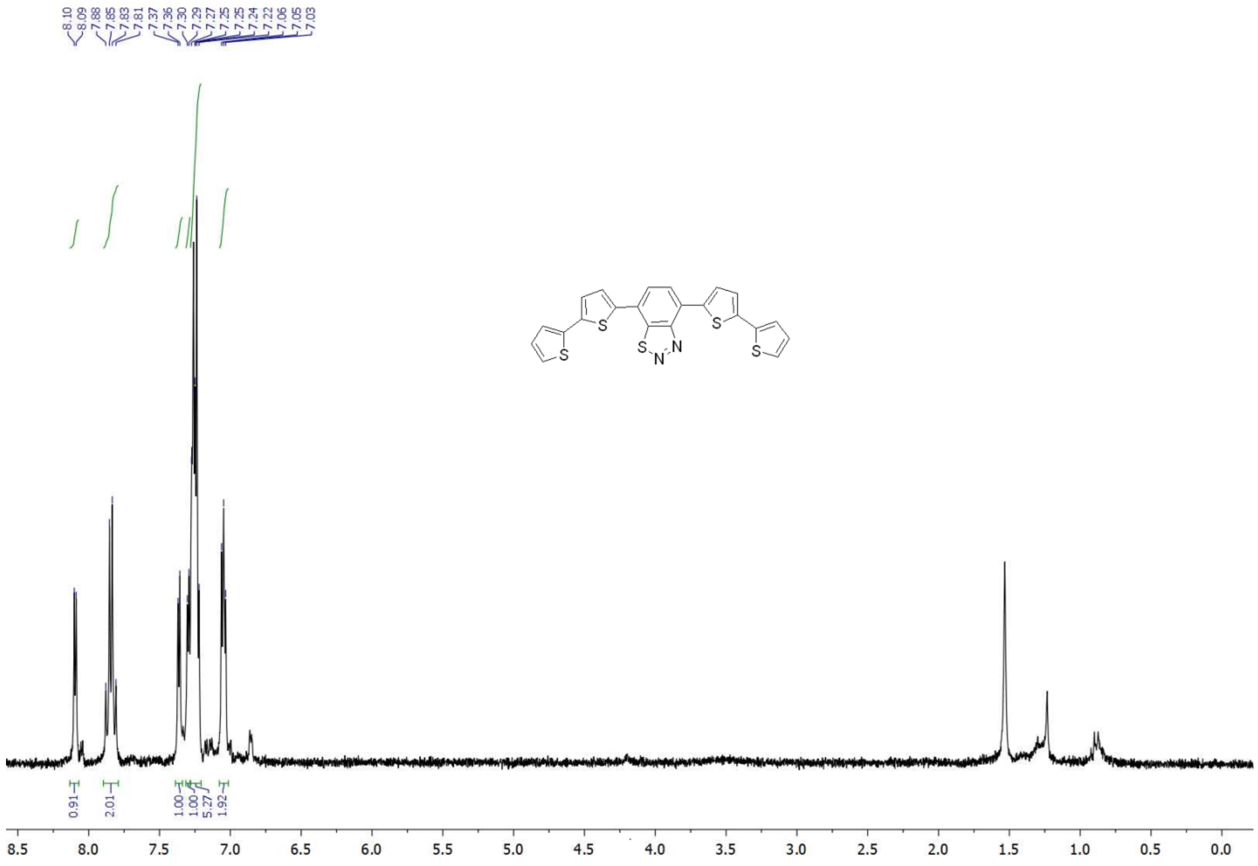

^{13}C NMR (75 MHz)

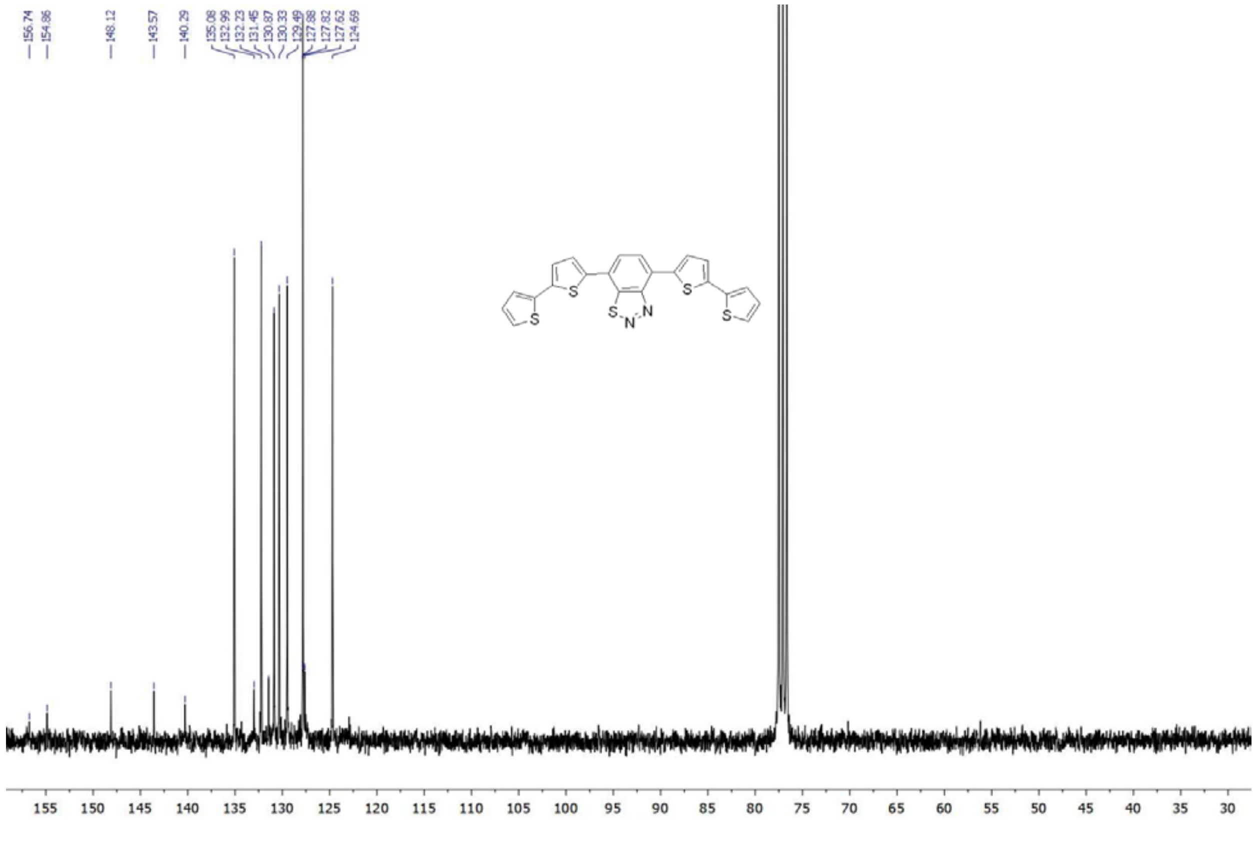

4,7-bis(2,3-dihydrothieno[3,4-b][1,4]dioxin-5-yl)benzo[d][1,2,3]thiadiazole (3e)

^{1}H NMR (300 MHz)

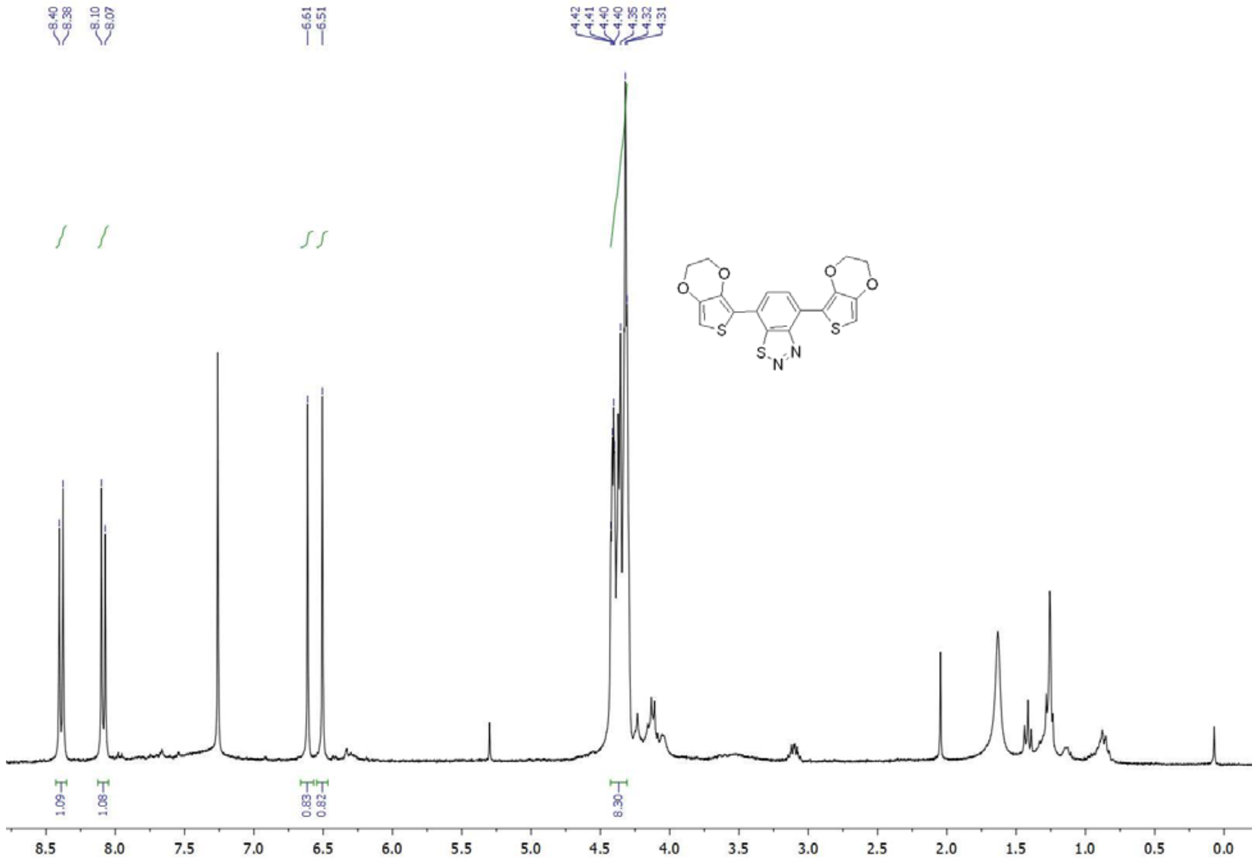

^{13}C NMR (75 MHz)

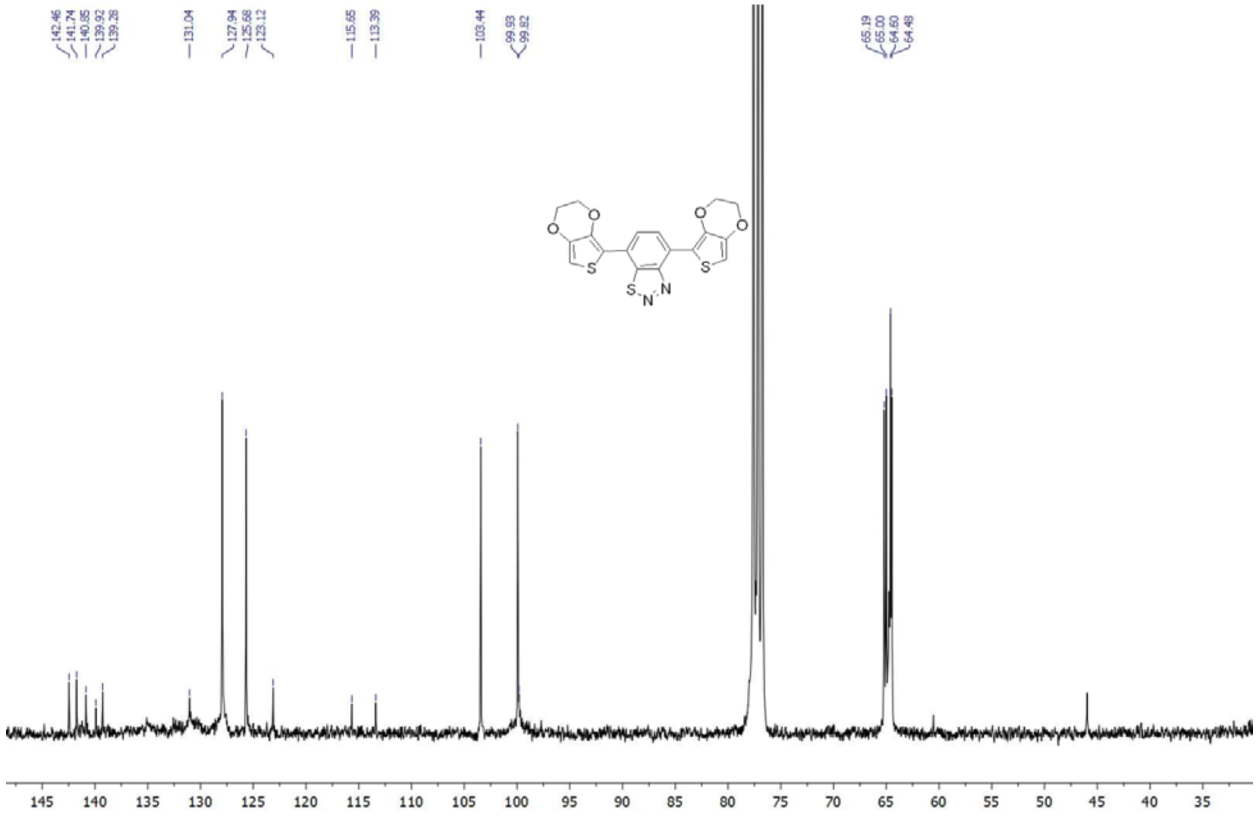

4-(4,4-bis(2-ethylhexyl)-4H-cyclopenta[2,1-b:3,4-b']dithiophen-2-yl)-7-(7,7-bis(2-ethylhexyl)-7H-cyclopenta[1,2-b:4,3-b']dithiophen-2-yl)benzo[d][1,2,3]thiadiazole 3(f)

^{1}H NMR (300 MHz)

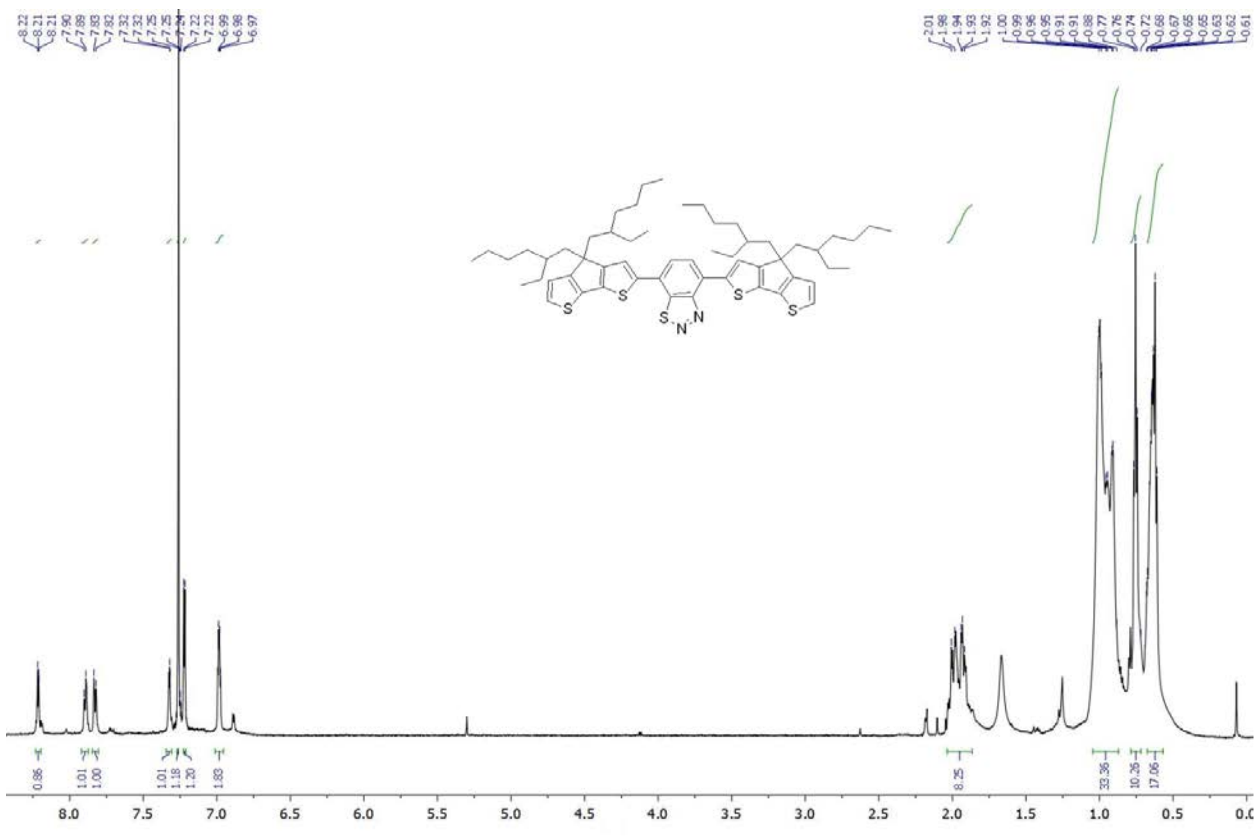

^{13}C NMR (75 MHz)

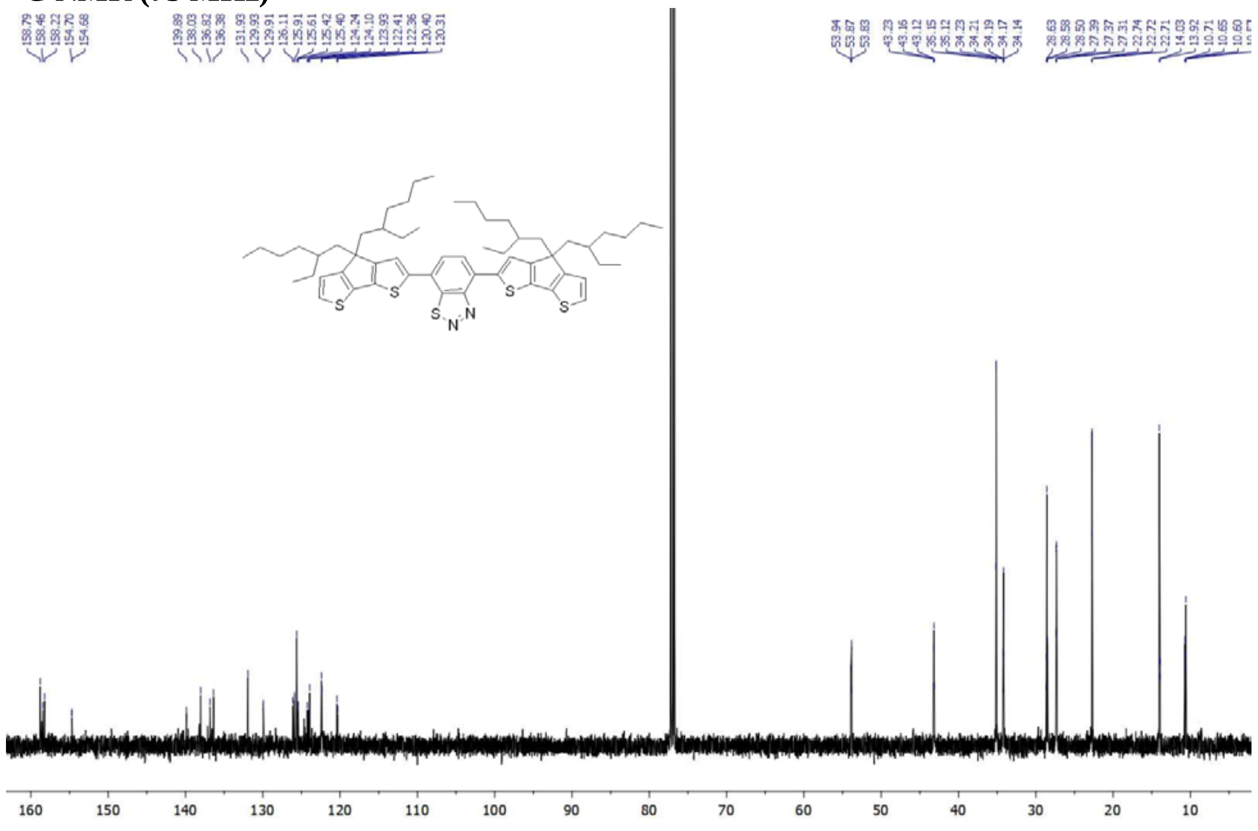

2. DFT calculations

DFT calculations were performed with the Gaussian 16 Rev C.01. M11 DFT functional with 6-31+g(d) basis set was used for all calculations. Calculations were performed in dichloromethane (PCM model). Data from various X-ray diffraction experiments were used as starting points for geometry optimizations. Cartesian coordinates are given in angstroms; absolute energies for all substances are given in hartrees. Analysis of vibrational frequencies was performed for all optimized structures. All compounds were characterized by only real vibrational frequencies. Wavefunction stability, using *stable* keyword, was also checked for each molecule.

For calculations of optimized geometries, frequencies, thermodynamics and MO energies following keywords were used:

# opt freq 6-31+g(d) scrf=(solvent=dichloromethane) nosymm m11

Benzo[d][1,2,3]thiadiazole

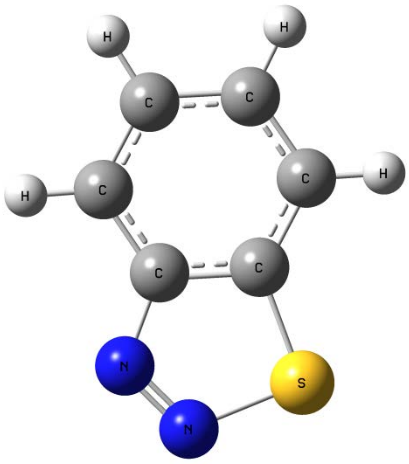

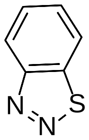

Charge 0; multiplicity 1

N	8.09236500	7.36061900	8.50926900
C	7.84273300	3.99915800	10.02589100
C	6.14424200	5.48723100	10.95643600
C	8.33577500	4.99246100	9.20557000
C	7.72294600	6.25541700	9.26135100
C	6.75464300	4.24813200	10.89377600
C	6.64353400	6.50178100	10.12276900
H	9.17350500	4.82232100	8.52910600
H	5.30596000	5.66947300	11.62820900
H	8.29507000	3.00700100	10.00811100
H	6.38709000	3.44200800	11.53060300
N	7.39644300	8.38840700	8.73075300
S	6.17509000	8.14385100	9.90675500

DFT M11/ 6-31+g(d) solvent dichloromethane, PCM model		
Total electronic energy=	-738.491320	E_0
Sum of electronic and zero-point Energies=	-738.400389	$E_0 + E_{ZPE}$
Sum of electronic and thermal Energies=	-738.394211	$E_0 + E_{tot}$
Sum of electronic and thermal Enthalpies=	-738.393266	$E_0 + H_{corr}$
Sum of electronic and thermal Free Energies=	-738.431247	$E_0 + G_{corr}$
Zero-point correction *(unscaled)* =	0.090931	
E HOMO, -9.45 eV		
E LUMO, -0.13 eV		
E gap, 9.32 eV		

HOMO:

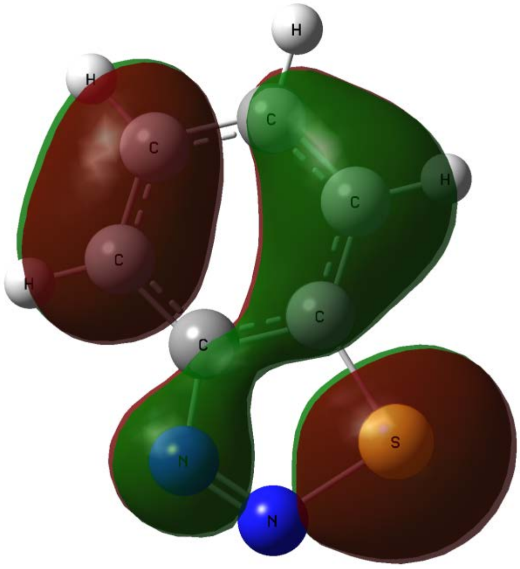

LUMO:

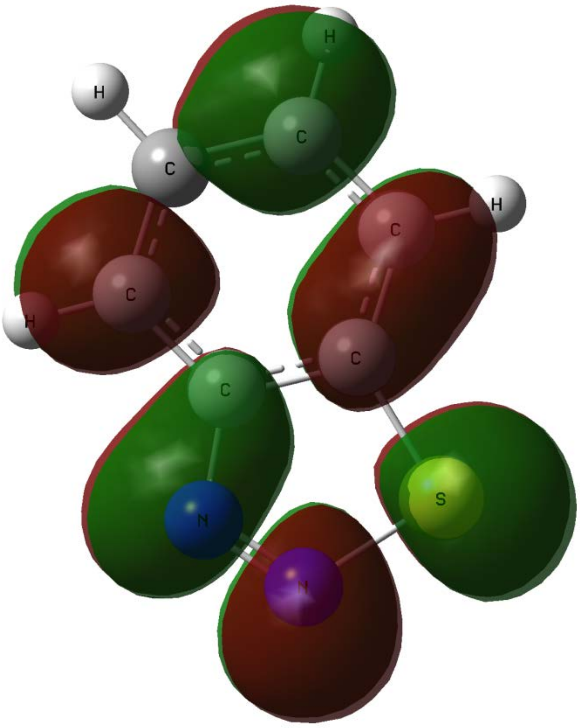

Benzo[c][1,2,5]thiadiazole

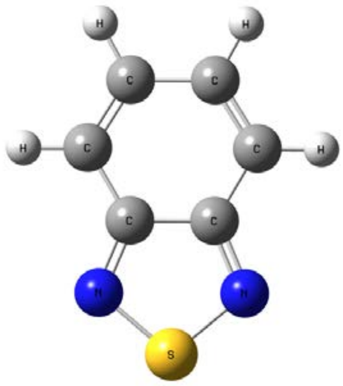

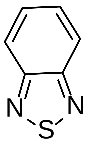

Charge 0; multiplicity 1

S	7.17487500	8.54737300	8.86790800
N	6.24587100	7.81089200	9.97437900
N	8.15379900	7.31714500	8.47284900
C	7.84242900	4.01434000	10.01884100
C	6.14992400	5.53300700	10.93371100
C	8.36937600	4.95922900	9.18880000
C	7.78381100	6.26347400	9.20578100
C	6.73309700	4.30113900	10.89076400
C	6.67561400	6.55036300	10.07766300
H	9.20863500	4.75043000	8.52572200
H	5.30986800	5.75793400	11.59052500
H	8.26616100	3.00942100	10.03027600
H	6.35593800	3.50311400	11.53138200

DFT M11/ 6-31+g(d) solvent dichloromethane, PCM model	
Total electronic energy=	-738.501331 E_0
Sum of electronic and zero-point Energies=	-738.410029 $E_0 + E_{ZPE}$
Sum of electronic and thermal Energies=	-738.403981 $E_0 + E_{tot}$
Sum of electronic and thermal Enthalpies=	-738.403037 $E_0 + H_{corr}$
Sum of electronic and thermal Free Energies=	-738.440767 $E_0 + G_{corr}$
Zero-point correction *(unscaled)* =	0.091302
E HOMO, -9.38 eV	
E LUMO, -0.71 eV	
E gap, 8.67 eV	

HOMO:

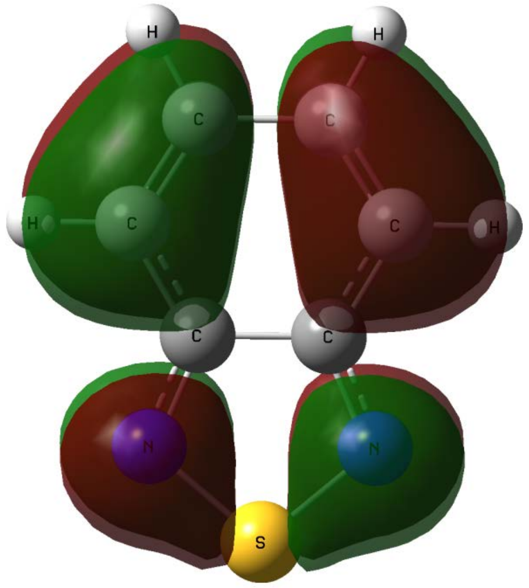

LUMO:

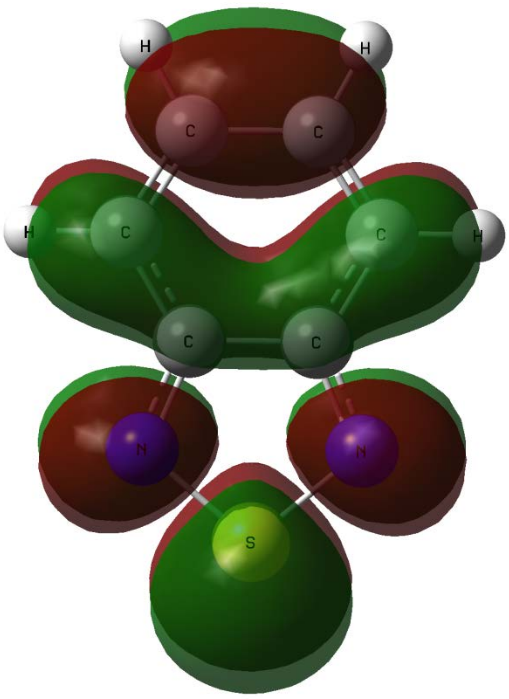

5,6-difluorobenzo[c][1,2,5]thiadiazole

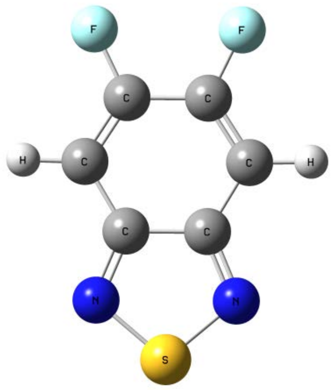

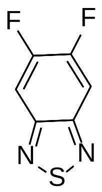

Charge 0; multiplicity 1

S	7.16425700	8.53998500	8.87136100
N	6.24376800	7.79661600	9.98063500
N	8.14730500	7.31368300	8.47252300
C	7.85729900	4.03750200	10.00665700
C	6.15215000	5.52875600	10.94330000
C	8.38713400	4.96262300	9.17469800
C	7.78621600	6.26066000	9.20386600
C	6.75088900	4.31776600	10.88179300
C	6.67869400	6.54162200	10.08097000
F	6.33693500	3.29099900	11.65016100
F	8.34527000	2.78255800	10.06153500
H	9.22480300	4.73033700	8.51980200
H	5.31558300	5.71989400	11.61270300

DFT M11/ 6-31+g(d) solvent dichloromethane, PCM model	
Total electronic energy=	-936.941583 E_0
Sum of electronic and zero-point Energies=	-936.866853 $E_0 + E_{ZPE}$
Sum of electronic and thermal Energies=	-936.859081 $E_0 + E_{tot}$
Sum of electronic and thermal Enthalpies=	-936.858137 $E_0 + H_{corr}$
Sum of electronic and thermal Free Energies=	-936.900090 $E_0 + G_{corr}$
Zero-point correction *(unscaled)* =	0.074730
E HOMO, -9.75 eV	
E LUMO, -0.89 eV	
E gap, 8.87 eV	

HOMO:

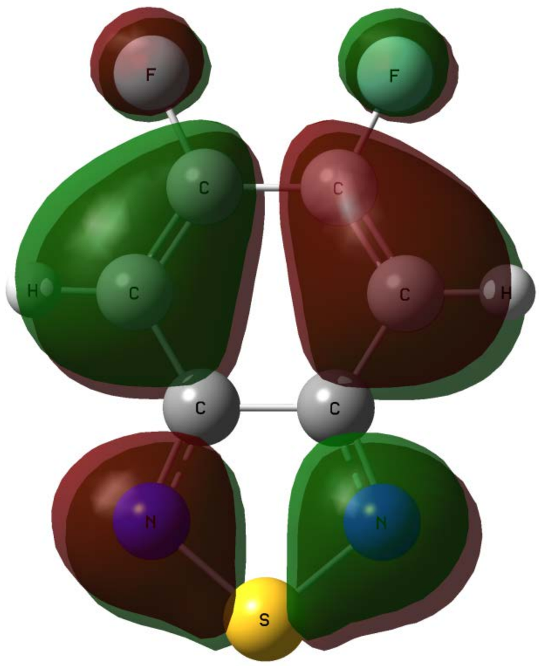

LUMO:

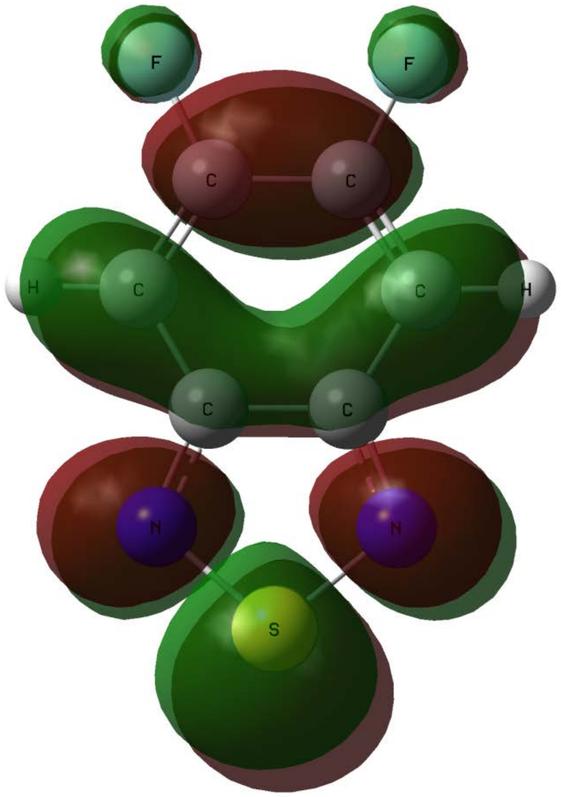

[1,2,5]thiadiazolo[3,4-c]pyridine

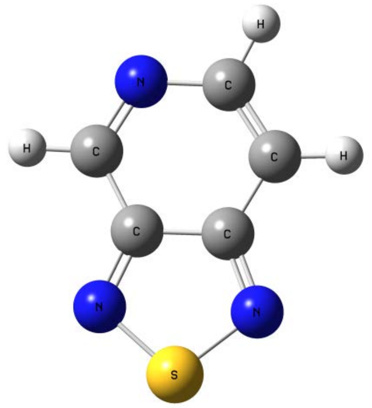

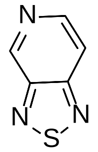

Charge 0; multiplicity 1

S	-0.43390300	6.73815300	4.90136100
N	0.37067100	7.38876800	6.14888200
N	-0.28548200	5.16881700	5.25916600
C	0.79490000	6.37532100	6.91084700
C	1.54728500	6.42464800	8.12044900
C	1.85216100	5.22960800	8.70274100
H	2.42575100	5.20061200	9.62985500
N	1.48881400	3.99176600	8.20965600
C	0.80153000	3.91697000	7.10703900
C	0.41448200	5.09776900	6.39471400
H	1.86079000	7.37327100	8.55340400
H	0.51965700	2.93196800	6.72666900

DFT M11/ 6-31+g(d) solvent dichloromethane, PCM model	
Total electronic energy=	-754.539108 E_0
Sum of electronic and zero-point Energies=	-754.459470 $E_0 + E_{ZPE}$
Sum of electronic and thermal Energies=	-754.453557 $E_0 + E_{tot}$
Sum of electronic and thermal Enthalpies=	-754.452613 $E_0 + H_{corr}$
Sum of electronic and thermal Free Energies=	-754.490164 $E_0 + G_{corr}$
Zero-point correction *(unscaled)* =	0.079637
E HOMO, -9.80 eV	
E LUMO, -1.05 eV	
E gap, 8.75 eV	

HOMO:

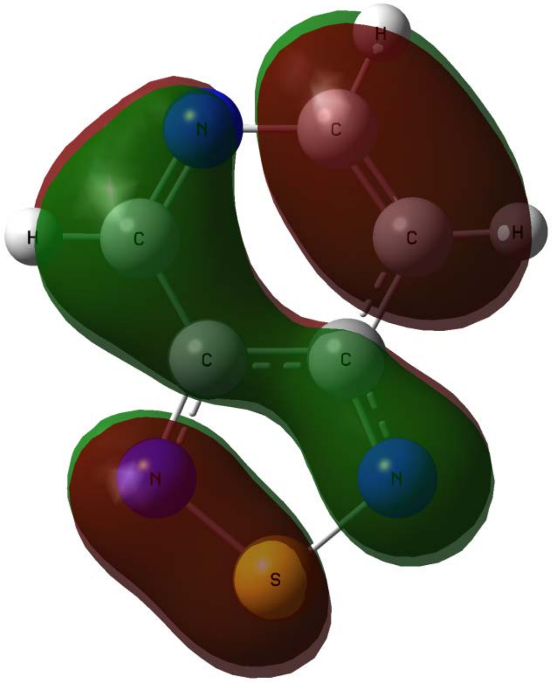

LUMO:

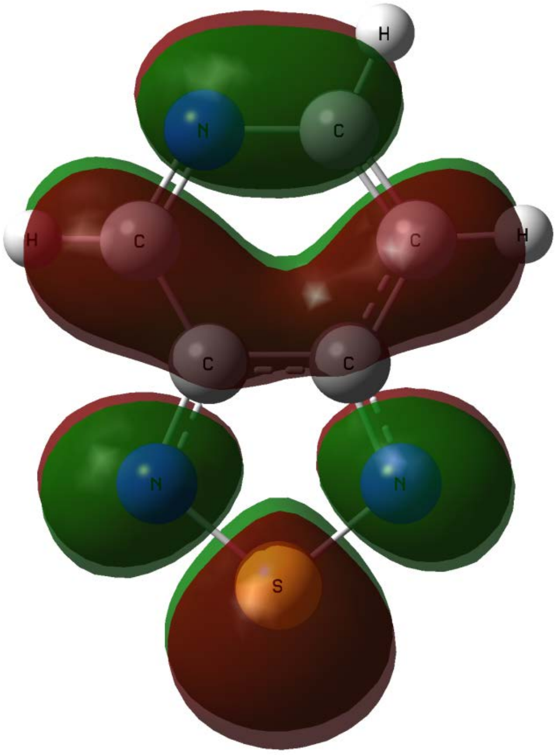

[1,2,5]thiadiazolo[3,4-d]pyridazine

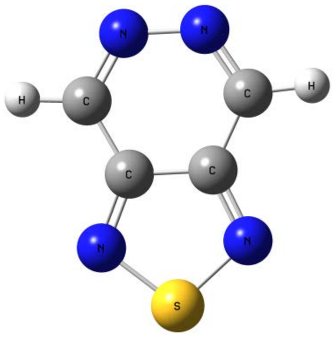

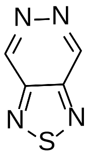

Charge 0; multiplicity 1

S	5.35843200	9.85373100	16.81976300
N	4.33676100	8.61341200	17.01394300
N	4.59259400	10.67115900	15.65141700
C	3.48618400	9.98056200	15.37028500
C	2.46085700	10.26608500	14.41372200
N	1.43664600	9.47886300	14.26614300
N	1.29437100	8.34083400	15.02094400
C	2.17816500	8.00433900	15.91334600
C	3.33971800	8.80625600	16.14853500
H	2.50816100	11.15319600	13.78002300
H	1.99951200	7.08676300	16.47638000

DFT M11/ 6-31+g(d) solvent dichloromethane, PCM model	
Total electronic energy=	-770.547491 E_0
Sum of electronic and zero-point Energies=	-770.480173 $E_0 + E_{ZPE}$
Sum of electronic and thermal Energies=	-770.474319 $E_0 + E_{tot}$
Sum of electronic and thermal Enthalpies=	-770.473375 $E_0 + H_{corr}$
Sum of electronic and thermal Free Energies=	-770.510886 $E_0 + G_{corr}$
Zero-point correction *(unscaled)* =	0.067318
E HOMO, -10.33 eV	
E LUMO, -1.34 eV	
E gap, 8.99 eV	

HOMO:

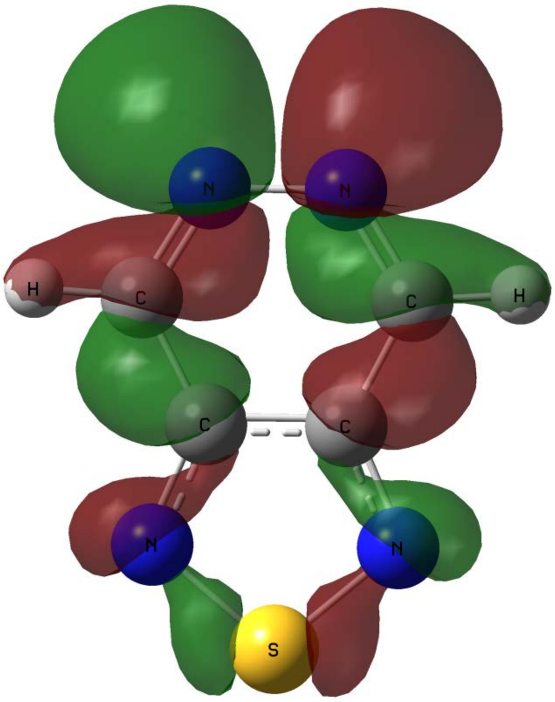

LUMO:

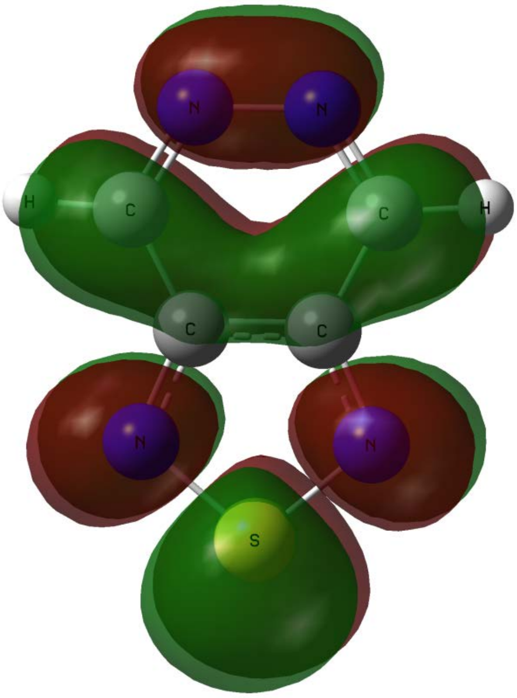

Benzo[1,2-c:4,5-c']bis([1,2,5]thiadiazole)

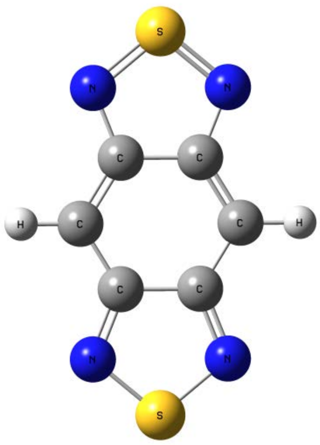

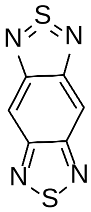

Charge 0; multiplicity 1

S	14.71994100	1.10676300	7.90031500
S	13.53694700	5.44711800	13.13946500
N	15.70196500	2.17699400	8.56746400
N	13.39170300	1.30697600	8.76644400
N	14.86509400	5.24704900	12.27322700
N	12.55487300	4.37704300	12.47219800
C	15.01058600	2.78977800	9.56373300
C	15.49824600	3.79337200	10.40129700
C	14.60545100	4.27659500	11.35838300
C	13.24608800	3.76457900	11.47559900
C	12.75827900	2.76127800	10.63779700
C	13.65120500	2.27781800	9.68093000
H	16.51354700	4.17606900	10.31336100
H	11.74289200	2.37877100	10.72549300

DFT M11/ 6-31+g(d) solvent dichloromethane, PCM model		
Total electronic energy=	-1244.88450	E_0
Sum of electronic and zero-point Energies=	-1244.803405	$E_0 + E_{ZPE}$
Sum of electronic and thermal Energies=	-1244.795603	$E_0 + E_{tot}$
Sum of electronic and thermal Enthalpies=	-1244.794659	$E_0 + H_{corr}$
Sum of electronic and thermal Free Energies=	-1244.837088	$E_0 + G_{corr}$
Zero-point correction *(unscaled)* =	0.081092	
E HOMO, -8.68 eV		
E LUMO, -1.99 eV		
E gap, 6.69 eV		

HOMO:

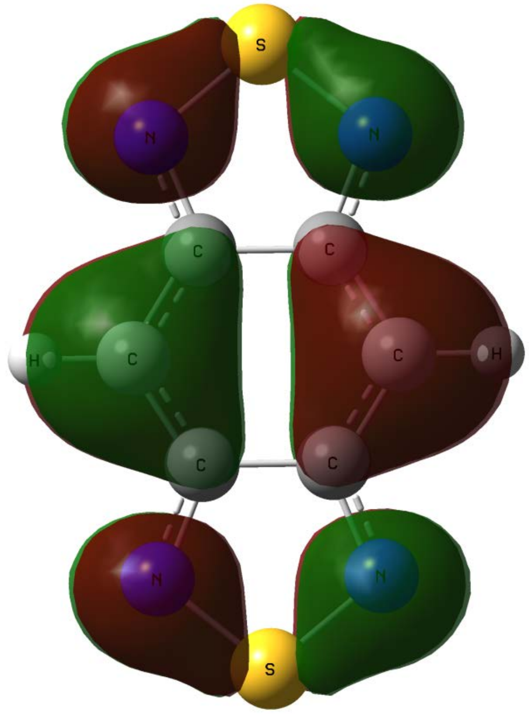

LUMO:

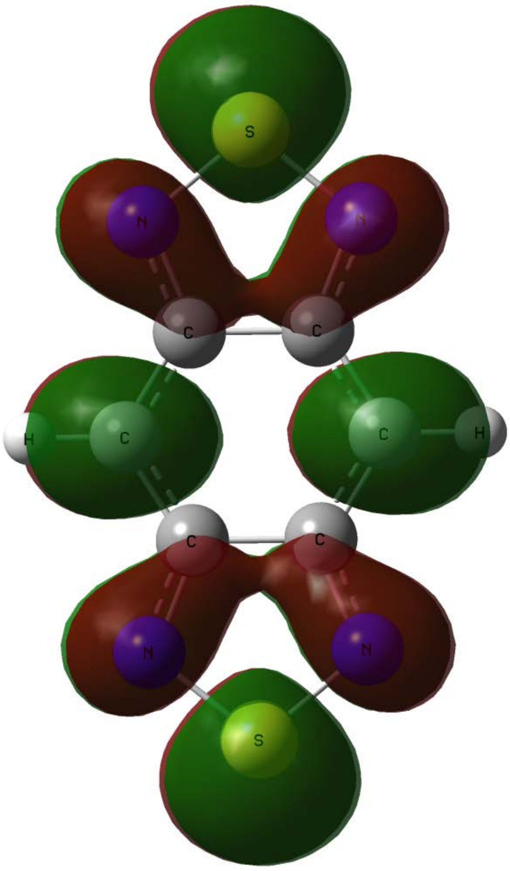

Supplement: Supplementary file 1 [file molecules-26-04931-s001.zip › molecules-1326398-supplementary/molecules-1326398-supplementary.pdf]
